# Model-Based Adaptive Machine Learning Approach in Concrete Mix Design

**DOI:** 10.3390/ma14071661

**Published:** 2021-03-28

**Authors:** Patryk Ziolkowski, Maciej Niedostatkiewicz, Shao-Bo Kang

**Affiliations:** 1 Faculty of Civil and Environmental Engineering, Gdansk University of Technology, Gabriela Narutowicza 11/12, 80-233 Gdansk, Poland; mniedost@pg.edu.pl; 2 School of Civil Engineering, Chongqing University, Chongqing 400045, China; kang0119@cqu.edu.cn; 3 Key Laboratory of New Technology for Construction of Cities in Mountain Area, Chongqing University, Chongqing 400045, China

**Keywords:** applied machine learning, concrete, concrete mix design, concrete strength prediction, data mining

## Abstract

Concrete mix design is one of the most critical issues in concrete technology. This process aims to create a concrete mix which helps deliver concrete with desired features and quality. Contemporary requirements for concrete concern not only its structural properties, but also increasingly its production process and environmental friendliness, forcing concrete producers to use both chemically and technologically complex concrete mixtures. The concrete mix design methods currently used in engineering practice are joint analytical and laboratory procedures derived from the Three Equation Method and do not perform well enough for the needs of modern concrete technology. This often causes difficulties in predicting the final properties of the designed mix and leads to precautionary oversizing of concrete properties for fear of not providing the required parameters. A new approach that would make it possible to predict the newly designed concrete mix properties is highly desirable. The answer to this challenge can be methods based on machine learning, which have been intensively developed in recent years, especially in predicting concrete compressive strength. Machine learning-based methods have been more or less successful in predicting concrete compressive strength, but they do not reflect well the variability that characterises the currently used concrete mixes. A new adaptive solution that allows estimating concrete compressive strength on the basis of the concrete mix main ingredient composition by including two observations for a given batch of concrete is proposed herein. In presented study, a machine learning model was built with a deep neural network architecture, trained on an extensive database of concrete recipes, and translated into a mathematical formula. Testing on four concrete mix recipes was performed, which were calculated according to contemporary design methods (Bolomey and Fuller method), and a comparative analysis was conducted. It was found out that the new algorithm performs significantly better than that without adaptive features trained on the same dataset. The presented algorithm can be used as a concrete strength checking tool for the concrete mix design process.

## 1. Introduction

Concrete mix is a mixture of cement, water, and fine-grained and coarse-grained aggregate, as well as additives and admixtures. Additives and admixtures are designed to improve the chemical characteristics and performance parameters of concrete, especially compressive strength, durability, and workability. One can distinguish many such supplements, including accelerating admixtures, admixtures for improved fresh concrete properties, durability-enhancing admixtures, fibers for concrete strengthening, set-retarding admixtures, and water-reducing admixtures. The concrete mix’s appropriate design is one of the most critical issues in the construction process, which is considered on many levels. The concrete mix must be designed economically. This means that it must allow obtaining the desired properties at the lowest possible cost of raw materials. A concrete mixture is dedicated to a specific technological process, where properties such as workability or cement setting speed are vital. Subsequently, there are environmental conditions related to precipitation, temperature during concreting, the distance from the construction site, and the traffic volume. The composition of the concrete mix significantly depends on the assumed construction specification resulting from the construction design, in which it is assumed, for example, the compressive strength of concrete, or environmental aggression, such as chloride ingression. The last important factor determining the composition of the concrete mix is ecological considerations, which have recently gained particular importance. There are currently many solutions to reduce the carbonation of concrete, such as admixtures of graphene nanoparticles [1].

In conclusion, it can be said that designing a concrete mix comes down to the appropriate selection of the proportions of the primary and secondary components in order to obtain concrete with the desired properties. During the technological process of concrete production, the concrete mix is transported to the construction site and placed in the concrete formwork; then, the process of concrete hardening and gaining strength occurs. The hydration of the cement initiates the concrete hardening process. Cement hydration is an exothermic chemical reaction that occurs when cement comes in contact with water. After starting the hydration process, the cement forms tobermorite gel, hydroxide, and other ingredients, which enhance adhesion between the fine-grained and coarse-grained aggregate. Throughout this process, hydration products continuously deposit on the cement grains and fill the area occupied by the water. The final stage of the hydration process is when all water molecules are bound, or there is no more unreacted cement. Hardened concrete obtains partial compressive strength a few days after the hydration process starts, and most of compressive strength after around 28 days (some types of concrete reach their full strength later) [2,3,4]. The amount of water needed to hydrate the cement entirely varies between 20% and 25% by weight, not including the water trapped in the pores [5,6]. In keeping with Power’s model, the water required to hydrate cement is 42% by weight [7,8]. Concrete mix design methods, which are currently used in engineering practice, derive from solutions developed over a dozen years ago and are based on an estimation of concrete mortar strength for bending [9,10,11]. The practical application of these methods is laborious and ineffective, and it does not consider the chemical complexity and variability of modern concrete mixtures.

The presented field needs new technological solutions that will face current challenges, and the answer might lay in machine learning-based methods that are more or less successful in predicting concrete compressive strength; however, they do not reflect well the variability of new chemically complex concrete mixes. This paper presents a novel adaptive approach that allows estimating concrete compressive strength on the basis of the concrete mix main ingredient composition by including two observations for a given batch of concrete. The proposed solution is a deep neural network with 46 hidden neurons, clustered in seven layers. The machine learning model was built, trained on an extensive database of concrete recipes, and translated into a mathematical formula. Testing on four concrete mix recipes was performed, which were also calculated according to the Bolomey and Fuller method, and a comparative analysis was conducted. The presented algorithm can be used as a concrete strength checking tool for the concrete mix design process. Machine learning is part of a rapidly developing field of knowledge, within the broader domain of artificial intelligence. It enables systems to automatically learn and improve from experience without direct programming and specific tasks. There are many different approaches to machine learning, among which the most popular are artificial neural networks (ANNs), with many branches such as deep neural networks (DNNs), recurrent neural networks (RNNs), and convolutional neural networks (CNNs). There are also other approaches, such as gene expression programming (GEP) and multi expression programming (MEP) [12,13,14]. The basic unit of an ANN is an artificial neuron, grouped into clusters. A single artificial neuron can be treated as a recitative information carrier, just like a human neuron excitably conducts an electrical signal. Artificial neurons in the network somewhat mimic the behavior of the human brain. The clusters of neurons are grouped into layers. The ANN typically includes at least three layers, an input layer, a hidden layer, and an output layer. The input and output layers consist of input neurons and output neurons, respectively. These neurons represent the variables that we want to introduce into the algorithm and the variables resulting from the algorithm’s execution. The neurons process the input data with the appropriate weight in the hidden layer and provide the output by the activation function. Depending on the type, specificity, amount of data, and machine learning architecture, hidden layers may function differently. In some methods, the input variable’s weight is assigned randomly, and, in others, the weight is meticulously calibrated by backpropagation. There is also a weight control mechanism, called the “learning rule”. It can be said that a greater number of hidden layers allows ANNs to solve more difficult problems, but more computing power and time will be needed for calculations. ANNs are taught how to solve a problem upon being fed examples. ANNs are proven effective in finding patterns that would be difficult for human recognition. A deep neural network (DNN) is an artificial neural network (ANN) with multiple layers of hidden neurons, and it is characterized by a high level of complexity [15,16,17,18].

## 2. Concrete Mix Design and Machine Learning

### 2.1. Contemporary Engineering Practice in Concrete Mix Design

Concrete mix design is a complex issue, often requiring extensive knowledge of concrete technology and vast experience. The main task in the design process is to select appropriate material compositions to obtain a concrete mix with desired properties, both in the form of a fresh mix during transport and concreting and in the form of hardened concrete. Specific properties can be expected at each stage of the concrete structure manufacturing process. Several features characterize concrete performance, such as plasticity, durability, compressive strength, and modulus of elasticity. The properties mentioned above have different priorities at different times; for example, adequate compressive strength is essential from the point of view of designed ultimate limit state, while adequate durability is essential in an aggressive environment [19,20,21,22]. Designing a mix with the improper specification can have many serious consequences; therefore, concrete mix producers fearing failure to meet the appropriate criteria often deliberately augment its parameters beyond designed values. This leads to the phenomenon called “concrete superstrength”, which on the surface may seem beneficial, because, for example, in a case of concrete compressive strength, the strength of concrete is higher; however, the disturbed stiffness of the structure may cause the structure to behave differently than envisaged by the designer [23].

Corporate engineering practice varies across the world, while also sharing significant similarities. In the European Union, the norm governing concrete technology issues is “EN 206 Concrete: Specification, performance, production, and conformity”, while the design of concrete structures is described in the standard “EN 1992-1-1: Eurocode 2: Design of concrete structures”. There are equivalents and national appendices for each standard, e.g., in Great Britain, the BS EN 206: 2013 + A1: 2016 standard is used, while, in Poland, the PN-EN 206 + A1: 2016-12 standard is used. Depending on the member state of the European Union, various methods of designing concrete mix are popular. In Poland, the methods of Bukowski, Eyman, Klaus, Kopycinski and Paszkowski are most often used, along with the so-called double coating method [24]. On the other hand, in the United States of America, Bolomey, Fuller, and the 0.45 power gradation chart methods are the most popular. Most of these methods are derived from the so-called “Three Equations Method”, a merged experimental–analytical approach [11,25,26]. The experimental–analytical approach means that we need to calculate the volume of ingredients needed by an analytical method and validate it using destructive laboratory tests. This method allows us to determine the amount of cement, water, and aggregate by weight per unit volume, using three equations of consistency, strength, and water-tightness. Consistency Equation (1) is incorporated into the water-demand formula, which helps to find the desired consistency.

(1) $$W=C\cdot w_{c}+K\cdot w_{k}\;[l],$$

where W is the amount of water in 1 m^3^ of concrete, expressed in units of volume (in this case, L), C represents the weight of cement in 1 m^3^ of concrete, expressed in kg, $w_{c}$ is a cement–water demand index which denotes the amount of water in 1 dm^3^ that should be added to 1 kg of a given class of cement, expressed in L/kg, K corresponds to the weight of aggregate in 1 m^3^ of concrete, expressed in kg, and $w_{k}$ is an aggregate–water demand index which denotes the amount of water in 1 dm^3^ that should be added to 1 kg of dry aggregate of a certain fraction to obtain the desired consistency, expressed in L/kg. The cement–water demand index and aggregate–water demand index depend on the grain size, shape, surface roughness, proportion in a given composition, and required consistency of the concrete mix. The water demand for concrete additives and admixtures is considered by adding it to aggregate or cement depending on the grain size. The cement–water and aggregate–water demand indices were developed by Stern and Bolomey [27,28]. The next equation is called the concrete compressive strength equation, which comes in two versions, Bolomey and Feret. This equation describes the relationship between the compressive strength of concrete and parameters such as the water–cement ratio and the grade of cement and aggregate. Equation (2) is the Feret version of the concrete compressive strength equation. Equation (3) is the Bolomey version of the concrete compressive strength equation.

(2) $$f_{cm}=A\left[\left(\frac{C}{W}+p\right)-a\right]\;[\mathrm{MPa}],$$

(3) $$f_{cm}=A_{1,2}\left(\frac{C}{W}\pm a\right)\;[\mathrm{MPa}],$$

where $f_{cm}$ is a medium concrete compressive strength, expressed in MPa, and  A, $A_{1,2}$ are coefficients that depend on the type and strength class of the aggregate and the strength class of the cement. The coefficient $A_{1}$ is taken when C/W < 2.5 and $A_{2}$ is taken when C / W > 2.5. C represents the weight of cement in 1 m^3^ of concrete, expressed in kg, W represents the amount of water in 1 m^3^ of concrete, expressed in L; p is the amount of air in 1 m^3^ of concrete, expressed in dm^3^, and a is a numerical value depending on the quality of cement and aggregate, and it can be taken as a constant equal to 0.5. The a value is positive when the water–cement ratio is greater than or equal to 2.5 and negative when the water–cement ratio is less than 2.5. The Feret equation is valid when the aggregate strength is lower than the grout strength and applies to porous concrete. Lastly, Equation (4) is called the water-tightness equation, which tells us that the volume sum of the individual components is equal to the entire concrete mix volume.

(4) $$\frac{C}{\rho_{c}}+\frac{K}{\rho_{k}}+W=1000\;[{\mathrm{dm}}^{3}],$$

where W refers to the amount of water in 1 m^3^ of concrete, expressed in L, C represents the weight of cement in 1 m^3^ of concrete, expressed in kg,$\;\rho_{c}$ is the cement density in kg/dm^3^, K is the weight of cement in 1 m^3^ of concrete, expressed in kg, and $\;\rho_{k}$ is the aggregate density in kg/dm^3^. The quantitative composition of the concrete mix, considered as the amount of cement, water, and aggregate in 1 m^3^ of mixture, can be calculated using the equations described above. The Three Equations Method has certain boundary conditions. The concrete mix porosity should not exceed 0.002 of the mix volume without air-entraining admixtures or 0.008 of the mix volume using air-entraining admixtures.

The entire process of concrete mix design consists of the following stages: determining the initial assumptions, determining the required properties of hardened concrete and fresh concrete mix, selecting and evaluating the components of the concrete mix, designing the mix composition, checking the technical characteristics of the fresh concrete mix and hardened concrete in a laboratory, and preparing a working recipe. During the formulation of initial assumptions, several factors need to be considered, such as the concrete mix’s intended use, which depends on properties of the newly designed structure, including the location, amount of reinforcement, and geometric characteristics of the cross-section. The primary technical characteristics of fresh concrete mix are the bulk density, consistency, and air content, whereas those for hardened concrete are the frost resistance, fire resistance, and class of concrete compressive strength. It is necessary to analyze the technological process, as well as assess the conditions of concrete maturation and the method of compacting fresh concrete mix. The concrete exposure class, which corresponds to the degree and type of environmental aggression and additional properties, such as concrete tightness, should also be specified. It is necessary to determine maximum aggregate diameter and mix workability. The concrete mix components should be selected and evaluated, including the proper type of cement, appropriate water, and aggregate quality, characterized by the relevant standards. After designing the concrete mix composition and laboratory tests, the last part of the process is preparing a working recipe for 1 m^3^ of concrete mix. It is also vital to consider the recipe changes that may result from the dampness of the aggregate and adapt it to individual conditions, such as the capacity of a transport vehicle [29,30].

### 2.2. Machine Learning in Prediction of Concrete Features

Machine learning is used in many science areas, from forecasting real-estate prices to identifying conditions on the basis of computed tomography images. One of these areas is civil and structural engineering. In civil and structural engineering, machine learning is used in structural health monitoring, crack detection, life-cycle cost analysis, prediction of soil compression coefficient, and many more. In forecasting concrete properties, research focuses on predicting a concrete’s compressive strength, which is one of the most critical parameters of concrete and defines its class.

The challenge in predicting concrete strength by machine learning was first described by Yeh et al. [31] in 1998. Using seven input variables, they performed ANN and linear regression to predict the strength of high-strength concrete. Yeh et al. trained their algorithm on many concrete samples, but they were not filtered in terms of content. Their analysis took into account the concrete samples in the maturing phase, even those that were 3 days old, which may have significantly distorted the results.

In 2003, the topic was developed further by Seung-Chang Lee [32]. He utilized a modular network architecture, which consisted of five ANNs with unique architectures. These unique architectures corresponded to concrete in different maturation phases up until achieving full concrete strength. To estimate the number of neurons in the input layer, he used the parameter condensation technique. Seung-Chang Lee claimed that the condensation and weighing techniques he used are useful in finding optimal network performance. However, since his ANN models the maturation process from the moment after pouring to reaching full strength, it has no practical application. From an engineering practice point of view, the focus should be on concrete that has reached its full or most of its strength.

In 2005, Hola J. and Schabowicz K. [33,34] presented an attractive nondestructive concrete strength assessment approach. They used an ANN model trained not on the concrete mix composition, but on the data collected by nondestructive concrete testing equipment. Their database contained ultrasonic wave velocity, reflection number, hardness, pull-out strength, concrete age, and bulk density. To obtain the laboratory results, they tested concrete compressive strength samples with a 28 day strength of 24–105 MPa. They created the ANN with eight hidden neurons grouped in one layer, using the Levenberg–Marquardt training method. The authors claimed that the average concrete compressive strength compared between the ANN and nondestructive tests was similar.

In 2006, Gupta et al. [35] proposed using a neural-expert system to predict the compressive strength of high-performance concrete. In their method, Gupta et al. focused on training the algorithm using example inferences and used a multilayer ANN trained with generalized backpropagation for interval training patterns. This may lead to algorithm training based on patterns with insignificant variables. They also used input variables of completely different metrics not strictly related to the recipe, such as curing time, and focused on basics such as the concrete mix composition, which may have an unclear effect on results. The neural-expert system in concrete compressive strength prediction was also discussed by Dac-Khuong Bui et al. [36], where they focused on the practical application of this approach.

In 2018, Fangming Deng et al. [37] introduced deep learning to the subject. Fangming Deng et al. for algorithm training purposes prepared a database of recycled concrete samples. Their database provided five input variables, such as fly ash replacement, recycled coarse-grained aggregate replacement ratio, recycled fine-grained aggregate replacement ratio, and water–cement ratio, used to train the machine learning algorithm. They decided not to train the algorithm on the concrete mix composition with a direct amount of individual components, but on the several ratios, which they referred to as deep features. In this study, a similar approach was used by introducing feature scaling. They used Softmax regression to look for a suitable prediction model. Fangming Deng et al. claimed that the introduction of deep learning compared to ANN provided better generalization capabilities, superior efficiency, and higher precision. However, this was not apparent and should be the subject of more extensive research. First, convolution neural networks are computationally expensive, as evidenced by the author’s adoption of a limited database. They used 74 samples in comparison to the 741 samples in this analysis. The limited number of samples may result in underfitting, which means that the model does not properly represent the modeled phenomenon. A similar level of accuracy between artificial neural networks and deep neural networks was presented by Hosein Naderpour et al. [38] in his study from 2018.

In 2019, Ziolkowski P. et al. [16] presented an algorithm, which supports designing a concrete mix by predicting the strength of concrete based on the composition of the concrete mix. The algorithm gave a quite right prediction of concrete mix strength. However, the paper’s algorithm gave a weak approximation for the high-strength spectrum of 40 MPa and above. It was also poorly able to predict the properties of mixtures with concrete additives and admixtures. Other essential parameters that contribute to proper concrete performance, such as durability, which is vital to maintain the service quality of structure in time, were not recognized in the study [39].

In 2020, Adil M. et al. [40] presented a paper in which they studied the effect of the number of neurons and layers in ANN for generalized concrete mix design. They used ANN with 17 inputs, such as the specific gravity of concrete mix ingredients, dry density of aggregates, type of cement and mineral admixtures, water–cement ratio, modulus of elasticity, and tensile and compressive strength of concrete, as well as five outputs, such as cement, water, and fine-grained and coarse-grained aggregate content. The authors argued that this network performed best with one or two hidden layers. It is an entirely different approach to a large number of previous works, where the technical parameters of concrete were predicted on the basis of the composition ratio.

In 2020, Nunez I. et al. [41] presented a study in which they built a machine learning model to predict the recycled aggregate concrete compressive strength and optimize the concrete mix design process. A reliable optimization method for concrete mix design is especially significant for recycled aggregate concrete, due to its variability and lack of proper compressive strength estimation formulas. The authors developed three distinctive machine learning models, namely, the Gaussian processes model, recurrent neural network model, and gradient boosted regression trees model, and they claimed to achieve robust predictive performance. They obtained the best performance using the gradient boosted regression trees model.

In 2020, Marani A. et al. [42] presented a solution to predict the compressive strength of ultra-high-performance concrete using a machine learning algorithm. They trained their algorithm on a database of 810 samples gathered from open-access sources. The database consisted of 15 variables that were taken as input data. The authors used an unusual technique, whereby, thanks to their database, they generated 6513 plausible synthetic data samples using tabular generative adversarial networks. Such a large pool of data allowed for robust training of their machine learning model. The authors found that their model trained on synthetic data achieved outstanding predictive performance when tested on the primary dataset.

## 3. Materials and Methods

### 3.1. Essentials

Most of the solutions described in the literature that could support the concrete mix design process consist of predicting the concrete compressive strength as a function of the concrete mix composition or basic technical parameters. The previously mentioned approaches could be improved by introducing an algorithm calibrated for a specific batch of concrete. This is essential for several reasons, the most important of which is the chemical complexity caused by various concrete admixtures, which is currently a standard. There are many different concrete admixtures, such as accelerating admixtures, admixtures for improved fresh concrete properties, durability-enhancing admixtures, fibers for concrete strengthening, set-retarding admixtures, water-reducing admixtures, and novel additions of nanomaterials such as graphene. These admixtures can significantly affect the obtained concrete parameters, especially compressive strength. It is also difficult to predict how a mixture with many different admixtures or admixtures produced by different manufacturers will behave in service. The second reason concerns the variability caused by the use of raw materials from different manufacturers, from different mining sources, and characterized by different properties. The variability can manifest itself on many levels, from the shape of the coarse aggregate to the presence of clay in the fine-grained aggregate. The algorithm’s primary task is to estimate concrete compressive strength as a function of the concrete mix composition calibrated by two observations. Observation encompasses the complete concrete recipe, along with compressive strength for a given type of concrete. The algorithm uses two recipes for a given concrete and tries to estimate the third recipe’s concrete compressive strength.

In current considerations, the database from a previous study [16] was used. The collected database serves as a basis for training the DNN with respect to the dependencies between individual input variables and the output variable. The data collection is extensive and includes various concrete mix recipes and laboratory tests results. The concrete mix recipes included in the database have been designed to be built into concrete structures of various dimensions, purposes, and functions. Some of them also contain admixtures of various origins and purposes, such as binding retardants, plasticizers, and workability boosters. Due to the abovementioned factors, some differences are challenging to predict between recipes of concrete mixes. Tested samples were standardized concrete cylinders with 15 cm diameter. Noncylindrical samples were converted into cylindrical following valid norms [43]. The samples were made from ordinary Portland cement. The aggregate size in the dataset did not exceed 20 mm. The parameters that were adopted are presented in Table 1.

The parameters presented in Table 1 are divided into two groups. The inputs refer to the input variables, such as cement, water, fine-grained and coarse-grained aggregate, and water–cement ratio. The target refers to the output variable, which is the concrete compressive strength. In these considerations, there is a general assumption that concrete achieves its designed compressive strength after 28 days. Such an assumption was made because, after the concrete mix fabrication, the cement hydration process begins, which progresses over time and increases the concrete’s strength until it reaches full strength. According to general knowledge, this process takes around 28 days (for some types of concrete, this time is longer). Before the indicated time, the concrete has partial strength. The samples in which the compressive strength test was performed earlier than 28 days were removed from the database. Table 2 presents the maximal, minimal, mean, median, and dominant value for each variable.

**Figure 1:**
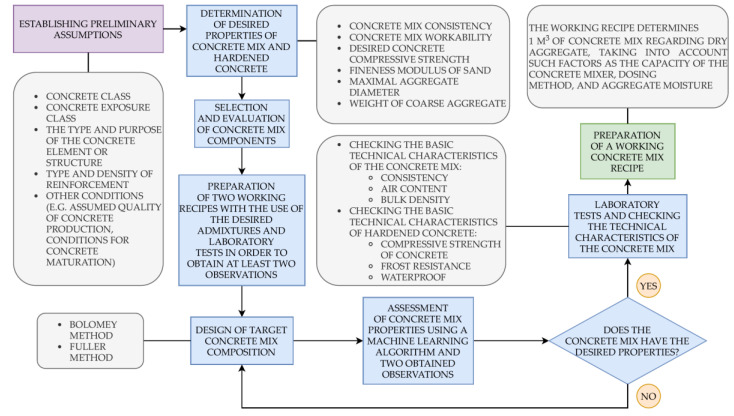
Flowchart presenting the practical application of the MAFM21 (Machine-learning Adaptive Forecasting Model 2021)-based formula in the concrete mix design process.

It is recommended to operate only within the limits set by range values for each input variable. Since the DNN is trained on a specific dataset, going beyond the range values can lead to the wrong results. The study did not directly analyze the effects of using con-crete additives and admixtures. Their influence is accounted for indirectly by including two observations if they affect the target variable. Many other processes influence hard-ened concrete properties, such as the curing process, but their influence was not consid-ered in this study. It is assumed that the quality control of the production of concrete mix and concrete was sufficient. As a result of this research, a trained DNN was obtained, translated into the source code, and interpreted as an equation, defining the 28 day com-pressive strength of concrete as a function of the 17 parameters. Of these, 12 parameters relate to the two recipes (cement, water, fine-grained and coarse-grained aggregate, water–cement ratio, and concrete compressive strength). The remaining five parameters describe the recipe for calculating the desired target value. A hyperbolic tangent was used for hid-den layers as the activation function and a linear tangent was used for the output layer. The practical application of the presented solution in the concrete mix design process is presented in Figure 1.

Later in this paper, the designed algorithm is referred to as MAFM21 (Ma-chine-learning Adaptive Forecasting Model 2021). In the comparative analysis presented below, we used the machine learning algorithm developed in the previous research [16]. This algorithm was built using a different model but was trained on the same dataset. It estimated the compressive strength of hardened concrete on the basis of the amount and composition of main concrete mix components. Later, this algorithm is referred to as MLM19 (Machine Learning Model 2019).

### 3.2. Results and Discussion

**Figure 2:**
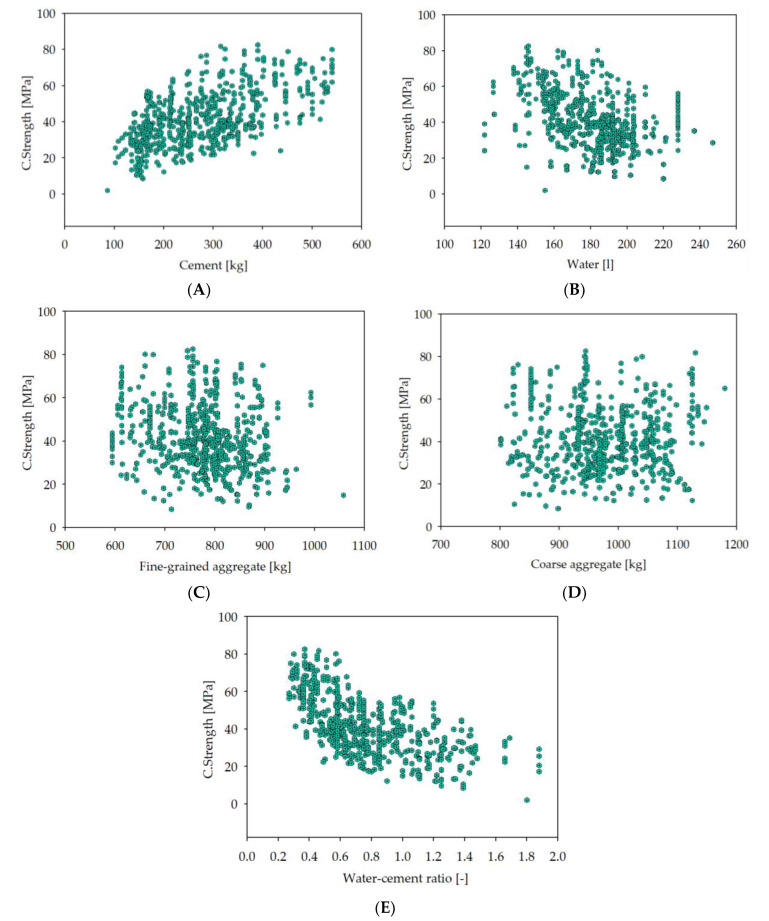
The scatter plots of target variable vs. input variables. The full compressive strength of concrete is on the vertical axis, expressed in MPa. The horizontal axis is the material content, expressed in kg for cement, fine-grained aggregate, and coarse-grained aggregate and L for water: (**A**) cement; (**B**) water; (**C**) fine-grained aggregate (sand 0–2 mm); (**D**) coarse-grained aggregate (aggregate above 2 mm); (**E**) water–cement ratio.

The dataset was divided into three subsets to perform DNN training, namely, training, selection, and testing datasets. These three datasets have distinct functions. The training dataset is used to feed the DNN, the selection dataset helps to adjust the hyperparameters of DNN, and the testing dataset serves as a tool to assess the DNN’s effectiveness. The whole database had 741 records, whereby the training dataset had 440 records (59%), the selection dataset had 146 records (20%), and the testing dataset had 146 records (20%). There were nine records (1%) excluded from the analysis as univariate outliers, with a maximum distance from the center of the dataset defined as three times the median value for each variable. This is a less conservative approach than n used in the previous study, where about 11% of the records were excluded. In Figure 2, scatter plots illustrating the concrete’s compressive strength related to the individual input variables are presented.

**Figure 3:**
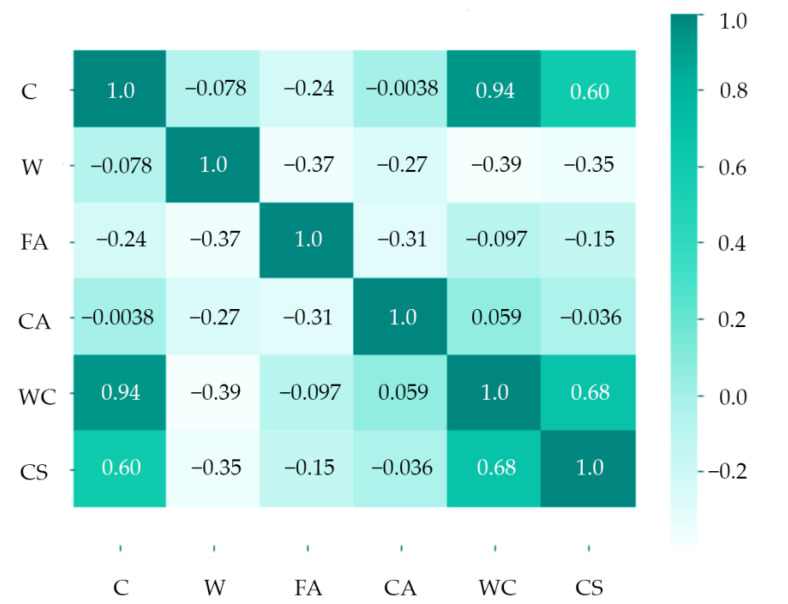
Feature correlation heatmap.

The initial DNN architecture comprised 17 input variables that refer to the 17 principal components and introduce one target output. The initial architecture contained three hidden layers, representing the complexity of the model. A DNN includes principal components, perceptron neurons, scaling neurons, and unscaling neurons. In the analysis, the feature scaling was used, hence the scaling and unscaling neurons. Two recipes were treated as observations by introducing into the equation a set of input variables representing cement, water, fine-grained and coarse-grained aggregate, water–cement ratio, and concrete compressive strength. These two recipes generated 12 input variables. The remaining five input variables corresponded to the composition of the third recipe for which it was tried to predict the concrete compressive strength. The input variables (cement, water, fine_grained_aggregate, coarse_aggregate, water_cement_ratio) presented in Table 1 corresponded with the input neurons. The target variable (cs_28) was associated with the output neuron. To find a proper training rate, the Broyden–Fletcher–Goldfarb–Shanno algorithm [44,45,46,47,48,49] was used. Next, to look for a quasi-Newton training direction, the Brent method [50,51,52,53] was applied. For analytical purposes, a linear correlation and a correlation matrix were assessed and calculated. Input contribution calculations were performed, where training inputs were selectively eliminated and the output results were inspected, whereby lower and higher input contribution values mean that the variable gave a lower or higher contribution to the results, respectively. The presented analysis indicated that the water–cement ratio had an immense contribution to the results. Since two observations were used with one actual recipe for predicting concrete compressive strength, every variable gave a slightly different contribution, which followed the overall pattern for each concrete mix ingredient. Feature correlation analysis was performed to show the relationship between the searched output variable of the compressive strength and the individual input variables, as presented in Figure 3. It can be seen that the water–cement ratio and the cement amount significantly impact the concrete strength. The significance of the water–cement ratio and the amount of cement for concrete compressive strength was also confirmed in literature by various researchers [54]. In these considerations, the main focus is on the effect of concrete mix composition on concrete compressive strength.

However, it should be noted that the strength of concrete is influenced by several other factors, primarily related to the environmental conditions and technological process of concrete production. The first important aspect is proper concrete curation after the built-in process. Improper concrete curation can significantly deteriorate the properties of concrete, especially its durability. Another vital issue is concreting during unfavorable weather conditions, mainly concrete freezing in the early stage of the hydration process and too high shrinkage due to drying during high temperatures. Considering environmental factors, one must refer to adverse environmental aggression, which harms the quality of concrete, for example, a high risk of carbonation [55] and chloride aggression in coastal regions. Concrete additives and admixtures also play an essential role in facilitating the technological process of concrete production and are widely used. The origin, shape, and texture of aggregate impact the workability and durability of concrete, but their influence is more significant on fresh concrete than hardened concrete [56]. Moreover, a grading and size distribution of aggregate determines the paste requirement for workable concrete [57]. In this analysis, several factors were not considered, such as the technological process, environmental factors, and raw material properties, assuming that the quality of obtained concrete samples was at an appropriate level, while all results deviating significantly from the mean values in the dataset were removed, treating them as univariate outliers.

**Figure 4:**
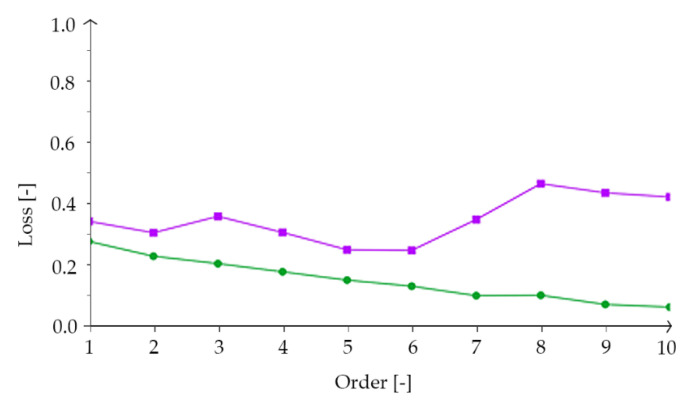
The loss history from incremental order algorithm execution, within 10 iterations, where the green line is the training loss and the purple line is the selection loss. Loss is on the vertical axis and order is on the horizontal axis.

The dataset had many input variables. Principal component analysis (PCA) was used to keep critical information while reducing input variables in a smaller feature space. Thanks to this method, among other things, it was possible to reduce the data dimensionality by feature extraction. The PCA uses auxiliary variables to explain most of the variability in the dataset [58,59,60,61,62]. One of the critical aspects when building an optimal DNN model is implementing the order selection algorithm. These algorithms help to minimize the loss of acquired data and find the most suitable DNN model with an optimal number of neurons which will match the data. In this study, an incremental order algorithm [63,64,65,66,67,68] was used. Figure 4 shows the training and selection loss history for various subsets from the incremental order algorithm performance within 10 iterations.

**Figure 5:**
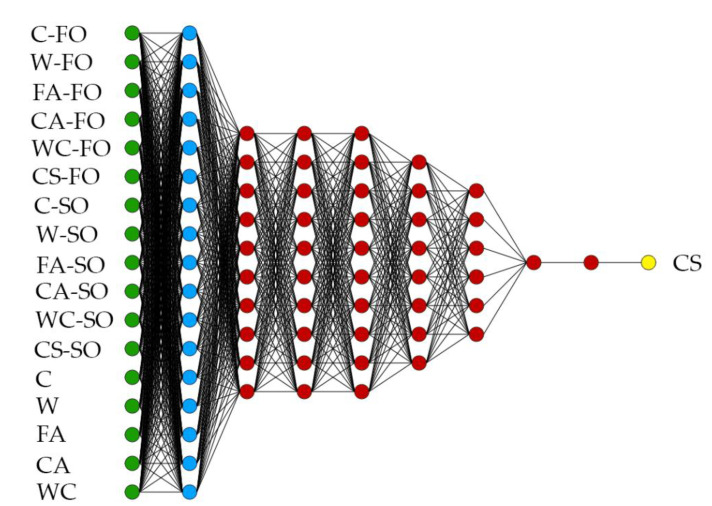
Final architecture of deep neural network (DNN) that was used. The figure shows the DNN architecture, which includes principal components (blue), perceptron neurons (red), and scaling and unscaling layers. The scaling neurons are green, and unscaling neurons are yellow. Abbreviations: C—cement; W—water; FA—fine-grained aggregate; CA—coarse-grained aggregate; WC—water–cement ratio; CS—the full concrete compressive strength; FO—first observation; SO—second observation.

The final architecture of the adopted DNN is presented in Figure 5. The final DNN model had seven hidden layers, 17 inputs, and one output. The model contained principal components (blue), perceptron neurons (red), scaling neurons (green), and unscaling neurons (yellow). The final DNN architecture should be the most optimal model for a given task.

The developed DNN had one target variable, the concrete compressive strength, with the 17 input variables representing two observation recipes and one recipe for the targeted value. Input variables expressed several concrete mix characteristics, such as cement, water, fine-grained and coarse-grained aggregate content, and water–cement ratio. The DNN was translated into a mathematical formula, presented as Equation (5), along with auxiliary Equations (A6)–(A49). Equation (5) refers to the 28 day strength of concrete, which can be considered as the full strength of concrete. The equation was optimized and simplified. The principal components were drawn into the equation.

(5) $$f_{c}^{full\;cs}=40.6152864-100.2590155\cdot \left(\tanh0.0630459-0.928083\cdot ax_{51}-2.03459\cdot ax_{52}+1.21399\cdot \;ax_{53}+1.17276\cdot ax_{54}-0.56805\cdot ax_{55}+0.191161\cdot ax_{56}\right)\;[\mathrm{MPa}].$$

The variables $C_{FO}$, $W_{FO}$, $FA_{FO}$, $CA_{FO}$, $CW_{FO}$, $CS_{FO}$, $C_{SO}$, $W_{SO}$, $FA_{SO}$, $CA_{SO}$, $CW_{SO}$, $CS_{SO}$, C, W, FA, CA, and WC used in Equations (A40)–(A49) denote the cement (C), water (W), fine-grained aggregate (FA), coarse aggregate (CA), water–cement ratio (WC), and concrete strength (CS). The first six variables represent the composition of the first observation recipe (FO). The subsequent six variables represent the composition of the second observation recipe (SO). The last five variables describe the actual concrete recipe for which the compressive strength was calculated. Units are expressed in kg/m^3^ or l/m^3^ for variables C, W, FA, and CA. Variable WC is a numerical ratio that is a dimensionless value. There were 36 auxiliary formulas, with Equations (A6) through (A39) containing variables with the index ax, which are the auxiliary variables presented in five layers, each with 6–10 variables. Equations (A6)–(A49) are presented in Appendix A.

The comparative analysis was prepared, which included a comparison of the MAFM21 algorithm with the MLM19 algorithm from 2019 presented in [16], which was trained on the same dataset but was built using a different model. Four concrete mix recipes were tested, designed according to the Bolomey and Fuller design methods, which are standard concrete mix design approaches. The first recipe was a standard mix consisting of cement, water, and fine-grained and coarse-grained aggregate with a plasticizer (lignosulfonate) and superplasticizer (polycarboxylate ether). The second recipe was a standard mix with plasticizer (lignosulfonate), superplasticizer (polycarboxylate ether), and air entrainer (tensides). The third was a standard mix with superplasticizer (polycarboxylate ether), retarder (phosphate), and air entrainer (tensides). The fourth was a standard mix with plasticizer (lignosulfonate) and superplasticizer (naphthalene). The recipes were prepared for 1 m^3^ of concrete designed as a concrete slab, with a plastic slump (which gives a lower value than the usual water–cement ratio), direct pouring with little environmental aggression, no special desired finishing, no special ambient conditions when casting, air content at 2.5%, and 20 mm maximum grain diameter. The following materials were adopted for the design: network water, ordinary Portland cement, natural sand, limestone gravel 4/10 mm, and limestone gravel 10/20 mm. Combinations of admixtures commonly used in engineering practice were selected. The exact composition of the designed mixtures is presented in Table 3 (Bolomey method) and Table 4 (Fuller method). Two similar recipes designed for a given compressive strength were used as observations to calculate the concrete compressive strength using the MAFM21 algorithm.

**Figure 6:**
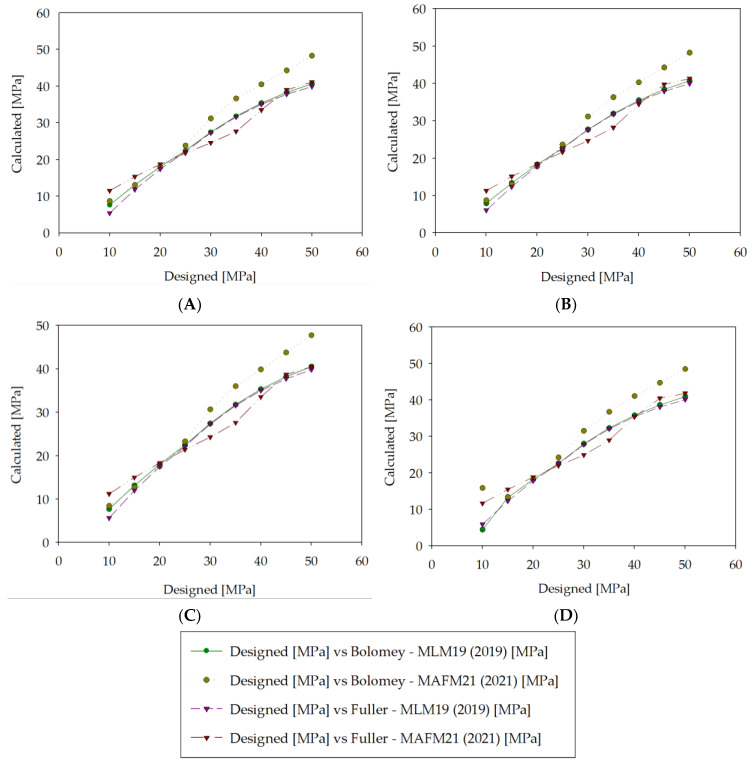
Comparison between designed concrete compressive strength and calculated concrete compressive strength according to MLM19 (Machine Learning Model 2019) and MAFM21 formulas for four recipes calculated according to the Bolomey and Fuller methods. (**A**) Recipe #1—standard mix + plasticizer (lignosulfonate) + superplasticizer (polycarboxylate ether); (**B**) Recipe #2—standard mix + plasticizer (lignosulfonate) + superplasticizer (polycarboxylate ether) + air entrainer (tensides); (**C**) Recipe #3—standard mix + superplasticizer (polycarboxylate ether) + retarder (phosphate) + air entrainer (tensides); (**D**) Recipe #4—standard mix + plasticizer (lignosulfonate) + superplasticizer (naphthalene).

Gradings and fitting curves for each designed concrete recipe are presented in Appendix B. The comparative analysis of errors is shown in Figure 6.

**Figure 7:**
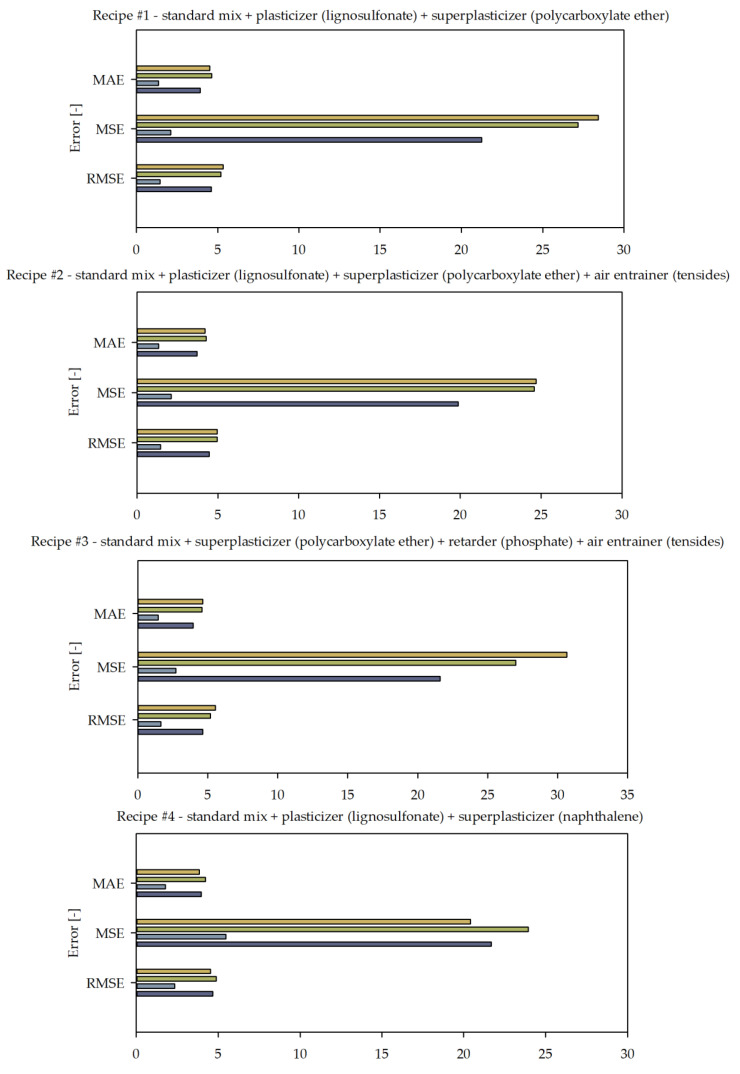

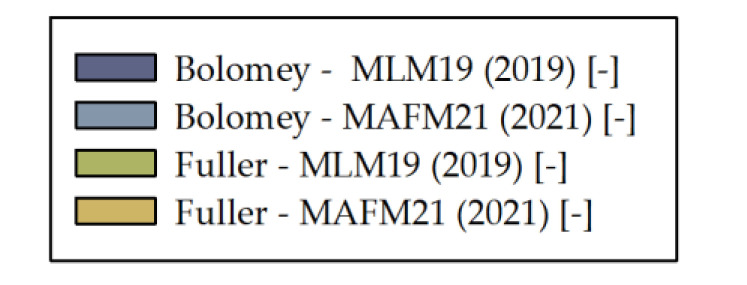
Errors in the comparison between designed concrete compressive strength and calculated concrete compressive strength according to MLM19 and MAFM21 formulas for four recipes designed according to the Bolomey and Fuller methods. MAE—mean absolute error; MSE—mean squared error; RMSE—root-mean-squared error.

The comparative analysis of errors is presented in Figure 7.

It can be seen in Figure 6 that the MAFM21 algorithm presented the best fit for mixtures designed according to Bolomey. Good convergence was especially visible for higher strength levels above 30 MPa. MAFM21 was also characterized as a good fit for mixtures designed according to Fuller in the lower ranges, below 20 MPa. However, there was some underestimation in the range from about 25 MPa to 40 MPa, depending on the mixture. It should also be noted that MAFM21 was more volatile than MLM19. For mixture number four, there was even considerable mismatch for 10 MPa. MLM19 had low resilience for high-strength recipes, whereas MAFM21 performed better in that spectrum. The statistical analysis of the errors presented in Figure 7 showed some interesting findings. Figure 7 shows that the MAFM21 algorithm was characterized by a similar level of mean absolute error (MAE), mean squared error (MSE), and root-mean-squared error (RMSE) for Fuller mixtures compared to MLM19 and significantly lower MAE, MSE, and RMSE values for Bolomey mixtures. Figure 7 presents almost a twofold decrease in MAE and RMSE for the Bolomey mix and a slight decrease in these errors for the Fuller mix for the first and second recipes. A significant difference can be noted looking at MSE, where, for the mixture according to the Fuller method, the error was on a similar level, whereas, for the mixture according to the Bolomey method, the error was five times smaller in favor of the MAFM21 algorithm. MAFM21 in the third recipe gave an even lower level of MAE, MSE, and RMSE for the Bolomey mixture; however, in this case, MLM19 had a lower MSE for the Fuller mix. Recipe four had a higher MSE for the MAFM21 Bolomey mix than the third recipe, but it was still significantly lower than the Bolomey mixture for MLM19. The MAFM21 algorithm for the fourth recipe also performed better in terms of MAE, MSE, and RMSE for Fuller mixes. It should be noted that the presented solution is still experimental, and it does not consider some key issues, such as the technological process and durability.

## 4. Summary and Conclusions

One of the most critical issues in the concrete structure manufacturing technological process is to obtain predictable properties of both fresh concrete mix and hardened concrete. Manufacturers of concrete mix are obliged to guarantee the appropriate properties of concrete delivered to the construction site. However, obtaining the appropriate concrete properties at all stages of the production process is difficult, especially when chemically complex additives and admixtures for concrete are present. Therefore, the properties of concrete mixes are often oversized, which can lead to very unfavorable phenomena. For example, increasing concrete strength more than the designed value may cause changes in the structure’s stiffness and lead to so-called concrete superstrength [23]. Predicting the properties of concrete is a complex issue, while most of the solutions used in engineering practice are approximate methods that, especially in recent years, due to the rapid development of materials engineering, have become outdated. Predictive analytics is an area of knowledge dealing with predicting all kinds of phenomena, properties, and trends. The most popular applications are economics, mathematics, and medicine. One of the most promising branches in predictive analytics is machine learning, which has acquired particular attention in recent years due to the significant development of technology. It could be used to significantly improve the process of concrete mix design.

The primary goal of this research was to introduce machine learning to the concrete mix design process and pave the way for new quality in this area of science. The MAFM21 algorithm can improve the ability to predict concrete technical parameters by including two observations. This adaptive approach allows us to predict the concrete mix behavior depending on its unique characteristics. Concrete mixes used in engineering practice have a very diverse composition. Designing a separate model for each specific composition of the concrete mix may turn out to be impractical and, in the long run, could be an obstacle to its widespread implementation. The core of the presented study was to develop an optimal deep neural network architecture and train it using an extensive database of concrete mix recipes with corresponding laboratory destructive tests. The developed algorithm estimates the concrete mix compressive strength according to its composition. The database used for training had 741 records, whereby the training dataset had 440 records (59%), the selection dataset had 146 records (20%), and the testing dataset had 146 records (20%). Nine records (1%) were excluded from the analysis as univariate outliners, with a maximum distance from the center of the dataset defined as three times the median value for each variable. The initially adopted DNN model had 17 input variables, 17 principal components, four hidden neurons, and one target output. The final DNN architecture had 17 input variables, 17 principal components, 46 hidden neurons, clustered into seven layers, and one target output. The applicable training rate and the step for the quasi-Newton training direction were calculated using the Broyden–Fletcher–Goldfarb–Shanno algorithm and the Brent method, respectively. The feature correlation analysis revealed that the most significant impact on concrete strength was the water–cement ratio and cement content. The dimensionality reduction was performed using principal component analysis. An incremental order algorithm was used to find the optimal number of neurons in DNN and minimize loss. The DNN source code was translated into a mathematical equation, which was then optimized and simplified. The final equation had 44 auxiliary equations. This method was tested on four concrete mix recipes, calculated according to contemporary design methods (Bolomey and Fuller method) and a comparative analysis of the new MAFM21 algorithm with the algorithm developed in 2019 MLM19 was conducted by analyzing the root-mean-squared error (RMSE), mean absolute error (MAE), and mean squared error (MSE). The conducted tests allowed us to see how MAFA21 complied with the methods used in engineering practice, and whether the adaptive approach performed better than the previous MLM19 algorithm trained on the same dataset.

The comparative analysis brought us a number of findings. The MAFM21 algorithm was more volatile, but had significantly lower RMSE, MAE, and MSE than MLM19 for mixtures designed according to the Bolomey method. MAFM21 gave slightly lower RMSE, MAE, and MSE for mixtures designed according to the Fuller method depending on the tested mixture. MAFM21 had the best fit for Bolomey mixes, characterized by particularly good convergence for higher strength levels above 30 MPa. MAFM21 performed well in lower ranges (below 20 MPa) for mixtures designed according to Fuller. MLM19 had low resilience for high-strength recipes, whereas MAFM21 performed better in that spectrum. The presented approach has boundary conditions and does not fully reflect all the relationships between the components of concrete mix and concrete properties. This issue requires further research. However, the results presented in this paper give hope for broader use of this method in engineering practice. Future research should extend the method’s use in the concrete mix design process by predicting other properties of fresh concrete mixtures and hardened concrete, such as durability, consistency, air content, and service life estimation. It would also be vital to create a more comprehensive approach for concrete mix optimization.

## Figures and Tables

**Table 1:** The parameters adopted in the dataset.

Parameter	Compressive Strength after 28 days	Cement	Water	Sand 0–2 mm	Aggregate above 2 mm	Water–Cement Ratio
Codename	cs_28	cement	water	fine_grained_aggregate	coarse_aggregate	water_cement_ratio
Type	Target	Input	Input	Input	Input	Input
Description	The 28 day compressive strength of concrete that is considered to have most of its strength.	Content of cement added to the mixture, expressed in kg.	Content of water added to the mixture, expressed in L.	Content of fine-grained aggregate added to the mixture, expressed in kg.	Content of coarse-grained aggregate with a size more than 2 mm, added to the mixture, expressed in kg.	Water-to-cement ratio.

**Table 2:** Value ranges of database input variables.

Input Variable	Minimum	Maximum	Mean	Median	Dominant
Cement	86.00 kg/m^3^	540.00 kg/m^3^	278.00 kg/m^3^	275.00 kg/m^3^	425.00 kg/m^3^
Water	121.80 kg/m^3^	247.00 kg/m^3^	182.42 kg/m^3^	185.00 kg/m^3^	192.00 kg/m^3^
Fine-grained aggregate (sand 0–2 mm)	372.00 kg/m^3^	1329.00 kg/m^3^	768.55 kg/m^3^	777.80 kg/m^3^	594.00 kg/m^3^
Coarse aggregate (aggregate above 2 mm)	597.00 kg/m^3^	1490.00 kg/m^3^	969.08 kg/m^3^	967.00 kg/m^3^	932.00 kg/m^3^
Water–cement ratio	0.27	1.88	0.76	0.69	0.45

**Table 3:** Tested concrete mix recipes designed according to Bolomey formula.

Designed Concrete Compressive Strength	Cement (kg/m^3^)	Water (L/m^3^)	Natural Sand (kg/m^3^)	Limestone Gravel 4/10 (kg/m^3^)	Limestone Gravel 10/20 (kg/m^3^)	Water–Cement Ratio (-)
Recipe #1—standard mix + plasticizer (lignosulfonate) + superplasticizer (polycarboxylate ether)						
10	177.09	57.56	1106.54	525.32	681.00	0.325
15	204.57	66.49	1059.83	531.08	674.29	0.325
20	232.05	75.42	1014.45	535.33	667.74	0.325
25	259.53	84.35	968.28	540.54	661.04	0.325
30	287.01	88.17	930.22	547.64	658.12	0.307
35	314.49	91.12	892.82	555.61	656.02	0.290
40	341.97	94.16	855.60	563.45	653.64	0.275
45	369.45	97.26	818.51	570.86	651.40	0.263
50	396.93	100.41	781.08	578.63	649.00	0.253
Recipe #2—standard mix + plasticizer (lignosulfonate) + superplasticizer (polycarboxylate ether) + air entrainer (tensides)						
10	178.26	57.93	1105.90	527.60	677.03	0.325
15	205.58	66.81	1060.69	532.11	670.39	0.325
20	233.33	75.83	1015.00	536.35	663.75	0.325
25	261.47	84.98	968.79	540.62	656.93	0.325
30	288.63	88.67	929.92	549.20	654.18	0.307
35	315.36	91.38	894.37	556.25	652.29	0.290
40	343.21	94.50	856.65	564.08	649.89	0.275
45	371.42	97.78	817.79	572.16	647.64	0.263
50	399.12	100.97	780.12	580.04	645.11	0.253
Recipe #3—standard mix + superplasticizer (polycarboxylate ether) + retarder (phosphate) + air entrainer (tensides)						
10	176.46	57.35	1107.69	526.38	677.27	0.325
15	203.84	66.25	1062.94	530.80	670.17	0.325
20	231.22	75.15	1016.61	536.10	663.75	0.325
25	258.60	84.05	971.48	540.13	657.40	0.325
30	285.99	87.86	932.98	548.07	654.22	0.307
35	313.37	90.80	896.19	555.60	652.09	0.290
40	340.75	93.82	859.54	562.61	650.09	0.275
45	368.13	96.92	821.81	571.15	647.48	0.263
50	395.51	100.05	784.51	578.91	645.11	0.253
Recipe #4—standard mix + plasticizer (lignosulfonate) + superplasticizer (naphthalene)						
10	179.00	58.17	998.39	369.05	947.31	0.325
15	206.77	67.20	1058.77	532.40	675.41	0.325
20	234.55	76.23	1012.64	537.16	668.59	0.325
25	262.32	85.26	966.36	542.02	661.83	0.325
30	290.10	89.12	925.90	547.94	652.76	0.307
35	317.88	92.10	890.13	553.89	650.72	0.290
40	345.65	95.17	850.22	564.03	648.42	0.275
45	373.43	98.31	812.49	571.92	646.00	0.263
50	401.20	101.49	774.79	579.60	643.63	0.253

**Table 4:** Tested concrete mix recipes designed according to Fuller formula.

Designed CC of Concrete Mix	Cement (kg/m^3^)	Water (L/m^3^)	Natural Sand (kg/m^3^)	Limestone Gravel 4/10 (kg/m^3^)	Limestone Gravel 10/20 (kg/m^3^)	Water–Cement Ratio (-)
Recipe #1—standard mix + plasticizer (lignosulfonate) + superplasticizer (polycarboxylate ether)						
10	177.09	57.56	1056.81	566.37	702.13	0.325
15	204.57	66.49	1035.14	554.76	687.74	0.325
20	232.05	75.42	1013.48	543.14	673.34	0.325
25	259.53	84.35	991.81	531.54	658.95	0.325
30	287.01	88.17	976.42	523.29	648.72	0.307
35	314.49	91.12	962.09	515.61	639.20	0.290
40	341.97	94.16	947.66	507.87	629.61	0.275
45	369.45	97.26	933.15	500.10	619.97	0.263
50	396.93	100.41	918.57	492.29	610.29	0.253
Recipe #2—standard mix + plasticizer (lignosulfonate) + superplasticizer (polycarboxylate ether) + air entrainer (tensides)						
10	178.26	57.93	1055.89	565.88	696.32	0.325
15	205.58	66.81	1034.35	554.33	682.12	0.325
20	233.33	75.83	1012.47	542.61	667.69	0.325
25	261.47	84.98	990.29	530.72	653.06	0.325
30	288.63	88.67	975.18	522.62	643.10	0.307
35	315.36	91.38	961.44	515.26	634.04	0.290
40	343.21	94.50	946.76	507.39	624.36	0.275
45	371.42	97.78	931.75	499.34	614.45	0.263
50	399.12	100.97	917.04	491.47	604.76	0.253
Recipe #3—standard mix + superplasticizer (polycarboxylate ether) + retarder (phosphate) + air entrainer (tensides)						
10	176.46	57.35	1057.31	566.64	697.26	0.325
15	203.84	66.25	1035.72	555.07	683.02	0.325
20	231.22	75.15	1014.13	543.50	668.79	0.325
25	258.60	84.05	992.55	531.93	654.55	0.325
30	285.99	87.86	977.20	523.71	644.44	0.307
35	313.37	90.80	962.93	516.06	635.02	0.290
40	340.75	93.82	948.55	508.35	625.54	0.275
45	368.13	96.92	934.09	500.60	616.00	0.263
50	395.51	100.05	919.57	492.82	606.43	0.253
Recipe #4—standard mix + plasticizer (lignosulfonate) + superplasticizer (naphthalene)						
10	179.00	58.17	1055.30	565.56	701.13	0.325
15	206.77	67.20	1033.41	553.83	686.59	0.325
20	234.55	76.23	1011.51	542.09	672.04	0.325
25	262.32	85.26	989.61	530.36	657.49	0.325
30	290.10	89.12	974.05	522.20	642.36	0.307
35	317.88	92.10	959.57	514.25	632.80	0.290
40	345.65	95.17	944.98	506.44	623.18	0.275
45	373.43	98.31	930.32	498.58	613.51	0.263
50	401.20	101.49	915.59	490.68	603.80	0.253

**Figure A1:**
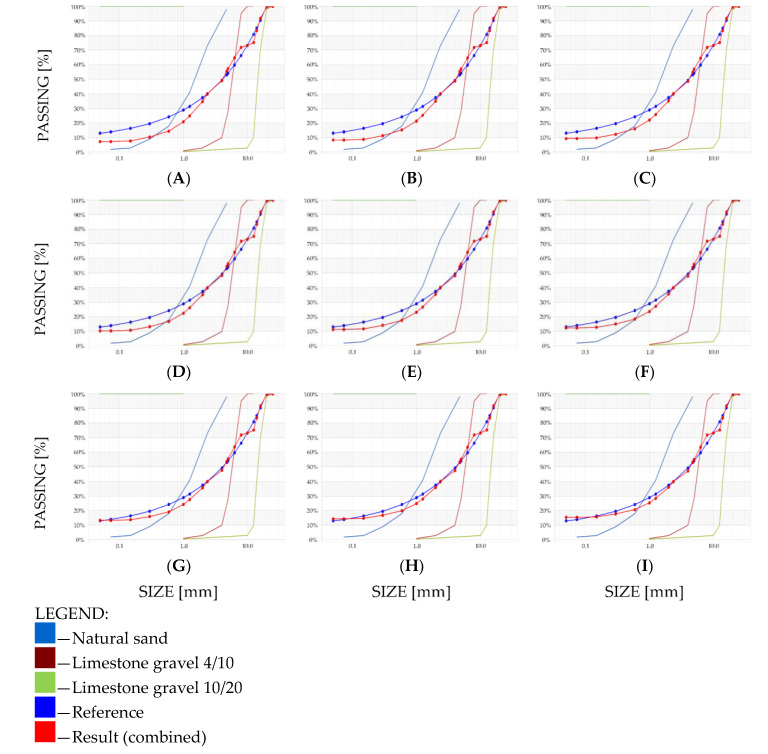
Gradings and fitting curves for Recipe #1—standard mix with plasticizer (lignosulfonate) and superplasticizer (polycarboxylate ether) designed according to the Bolomey Method: (**A**) 10 MPa; (**B**) 15 MPa; (**C**) 20 MPa; (**D**) 25 MPa; (**E**) 30 MPa; (**F**) 35 MPa; (**G**) 40 MPa; (**H**) 45 MPa; (**I**) 50 MPa. Legend in the left bottom corner.

**Figure A2:**
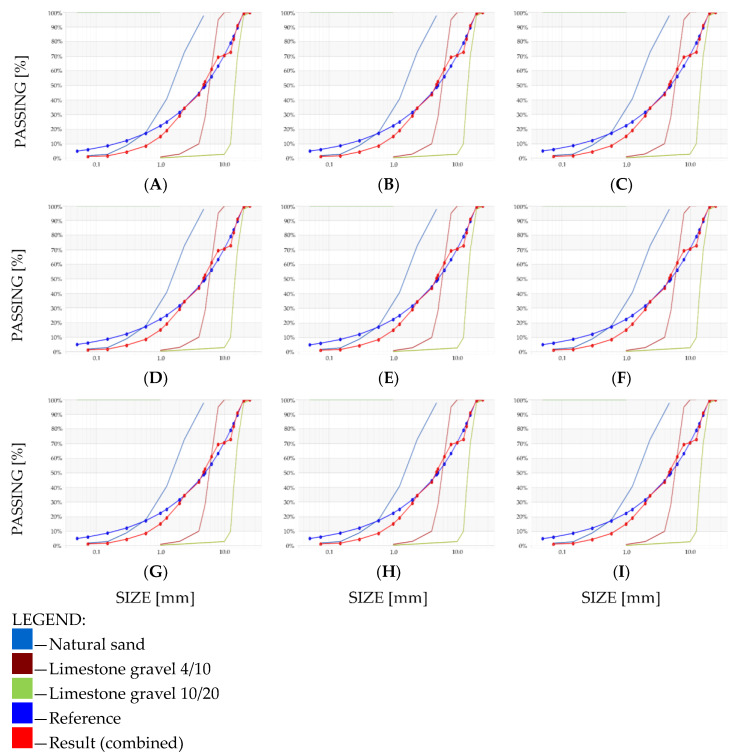
Gradings and fitting curves for Recipe #1—standard mix with plasticizer (lignosulfonate) and superplasticizer (polycarboxylate ether) designed according to the Fuller Method: (**A**) 10 MPa; (**B**) 15 MPa; (**C**) 20 MPa; (**D**) 25 MPa; (**E**) 30 MPa; (**F**) 35 MPa; (**G**) 40 MPa; (**H**) 45 MPa; (**I**) 50 MPa. Legend in the left bottom corner.

**Figure A3:**
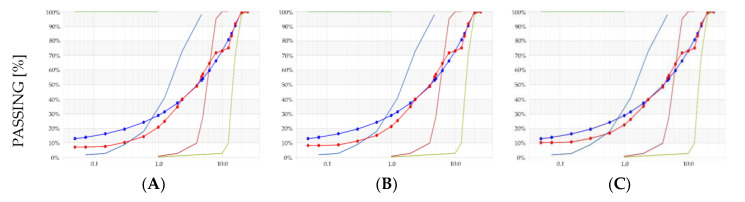

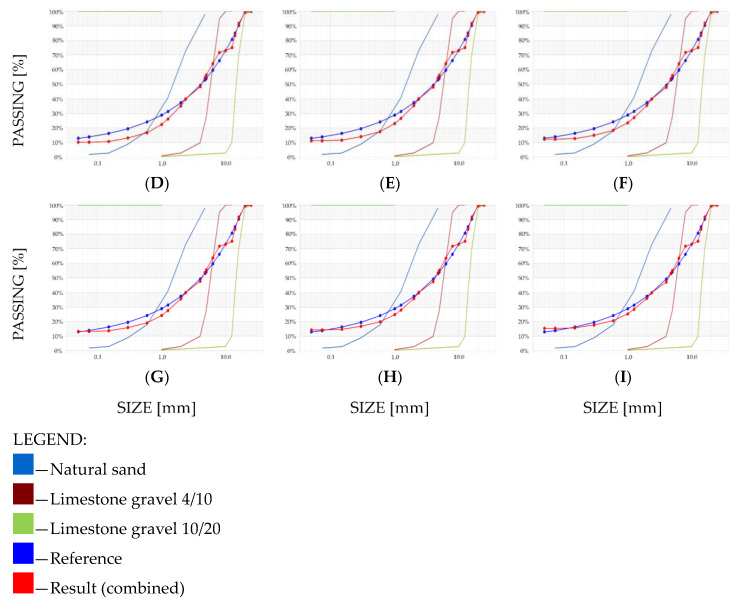
Gradings and fitting curves for Recipe #2—standard mix with plasticizer (lignosulfonate), superplasticizer (polycarboxylate ether) and air entrainer (tensides) designed according to the Bolomey Method: (**A**) 10 MPa; (**B**) 15 MPa; (**C**) 20 MPa; (**D**) 25 MPa; (**E**) 30 MPa; (**F**) 35 MPa; (**G**) 40 MPa; (**H**) 45 MPa; (**I**) 50 MPa. Legend in the left bottom corner.

**Figure A4:**
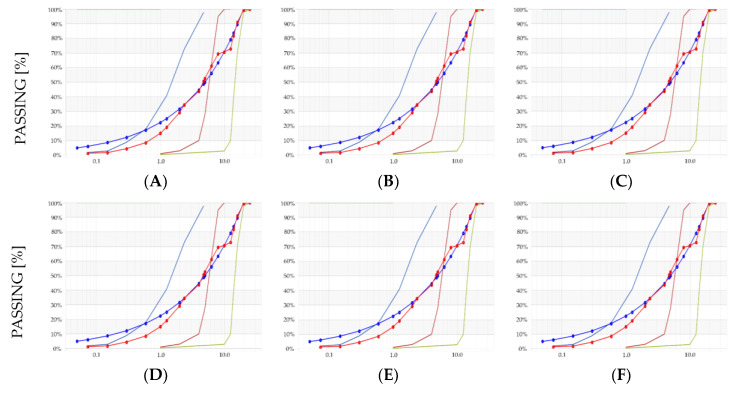

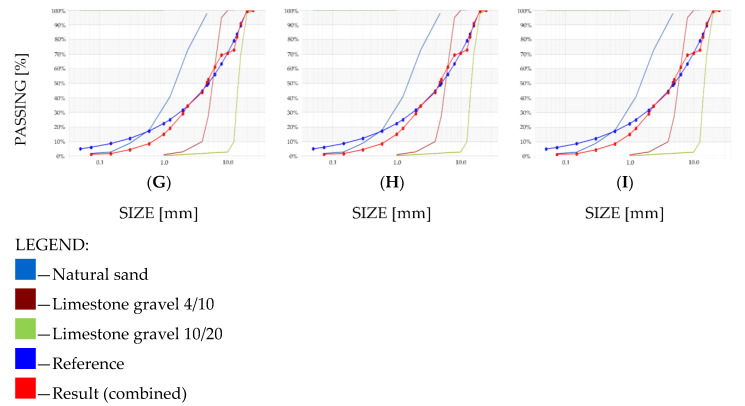
Gradings and fitting curves for Recipe #2—standard mix with plasticizer (lignosulfonate), superplasticizer (polycarboxylate ether) and air entrainer (tensides) designed according to the Fuller Method: (**A**) 10 MPa; (**B**) 15 MPa; (**C**) 20 MPa; (**D**) 25 MPa; (**E**) 30 MPa; (**F**) 35 MPa; (**G**) 40 MPa; (**H**) 45 MPa; (**I**) 50 MPa. Legend in the left bottom corner.

**Figure A5:**
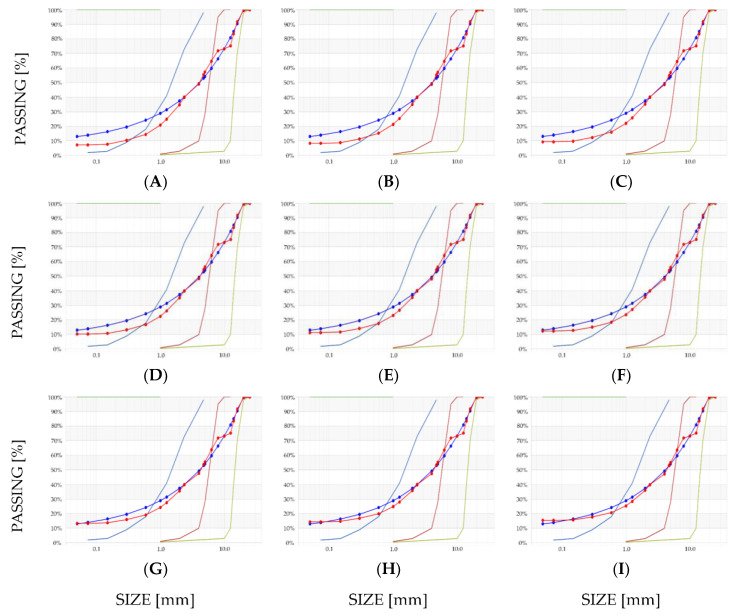

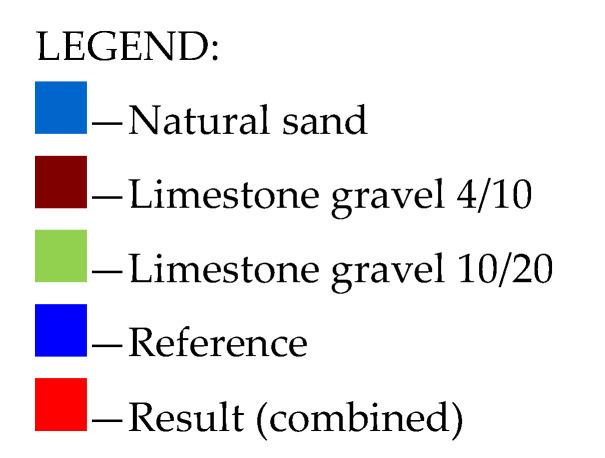
Gradings and fitting curves for Recipe #3—standard mix with superplasticizer (polycarboxylate ether), retarder (phosphate) and air entrainer (tensides) designed according to the Bolomey Method: (**A**) 10 MPa; (**B**) 15 MPa; (**C**) 20 MPa; (**D**) 25 MPa; (**E**) 30 MPa; (**F**) 35 MPa; (**G**) 40 MPa; (**H**) 45 MPa; (**I**) 50 MPa. Legend in the left bottom corner.

**Figure A6:**
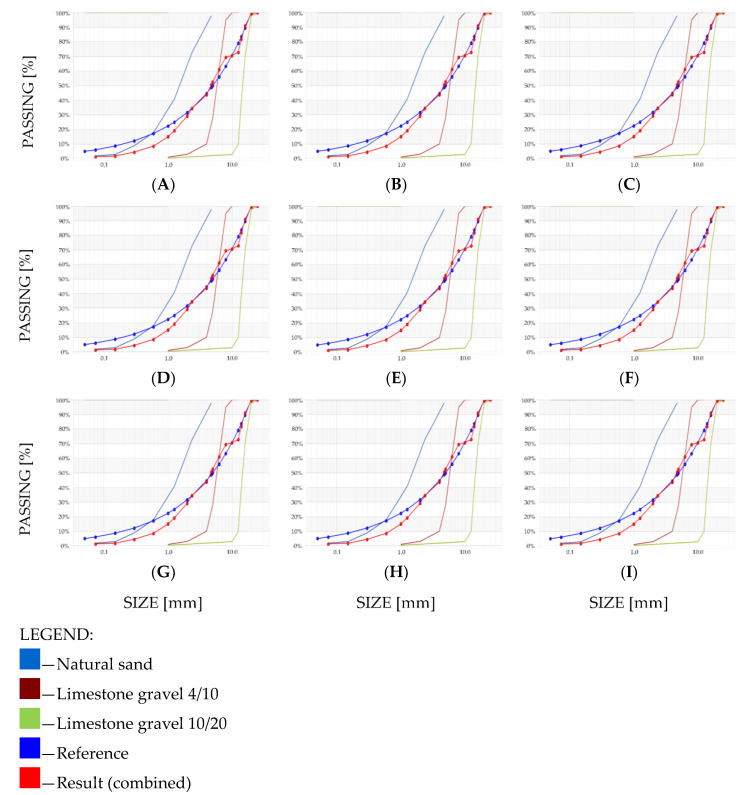
Gradings and fitting curves for Recipe #3—standard mix with superplasticizer (polycarboxylate ether), retarder (phosphate) and air entrainer (tensides) designed according to the Fuller Method: (**A**) 10 MPa; (**B**) 15 MPa; (**C**) 20 MPa; (**D**) 25 MPa; (**E**) 30 MPa; (**F**) 35 MPa; (**G**) 40 MPa; (**H**) 45 MPa; (**I**) 50 MPa. Legend in the left bottom corner.

**Figure A7:**
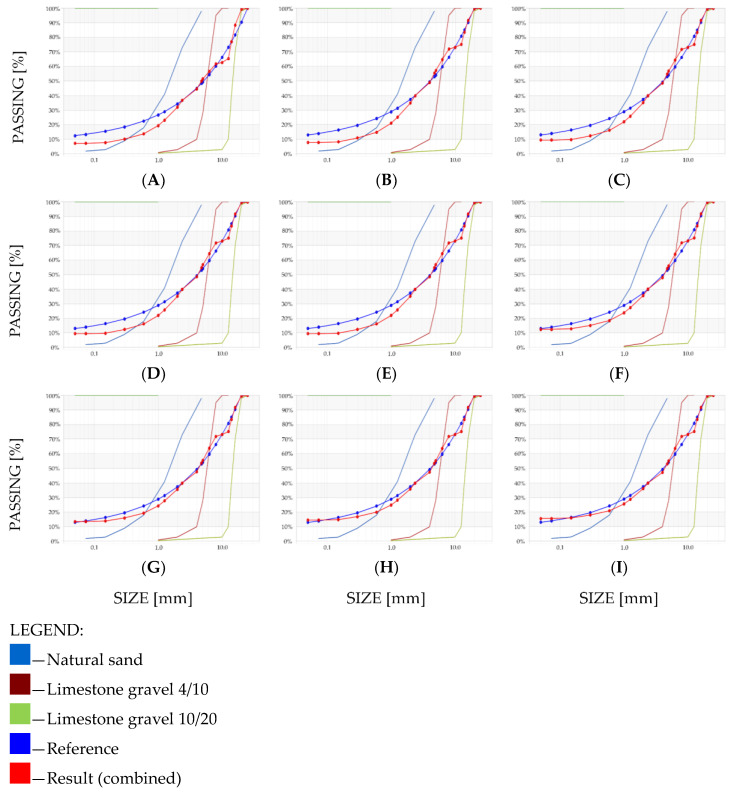
Gradings and fitting curves for Recipe #4—standard mix with plasticizer (lignosulfonate) and superplasticizer (naphthalene) designed according to the Bolomey Method: (**A**) 10 MPa; (**B**) 15 MPa; (**C**) 20 MPa; (**D**) 25 MPa; (**E**) 30 MPa; (**F**) 35 MPa; (**G**) 40 MPa; (**H**) 45 MPa; (**I**) 50 MPa. Legend in the left bottom corner.

**Figure A8:**
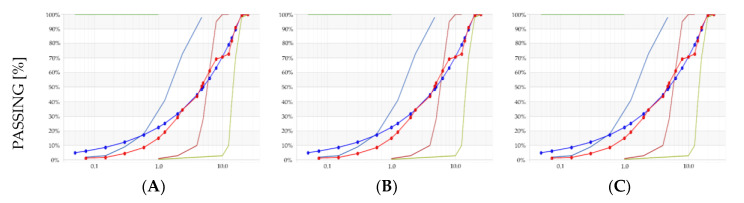

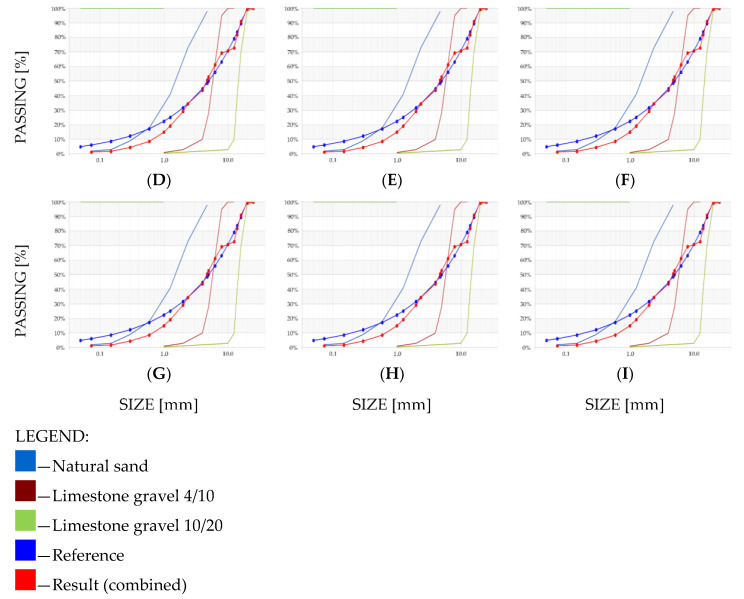
Gradings and fitting curves for Recipe #4—standard mix with plasticizer (lignosulfonate) and superplasticizer (naphthalene) designed according to the Fuller Method: (**A**) 10 MPa; (**B**) 15 MPa; (**C**) 20 MPa; (**D**) 25 MPa; (**E**) 30 MPa; (**F**) 35 MPa; (**G**) 40 MPa; (**H**) 45 MPa; (**I**) 50 MPa. Legend in the left bottom corner.

## Data Availability

No new data were created or analyzed in this study. Data sharing is not applicable to this article.

## Appendix A

Appendix A contains the auxiliary Equations (A6)–(A49) that are part of the main Equation (5).

(A6) $$ax_{51}=\tanh\left(-0.112106+0.345284\cdot ax_{41}+0.312406\cdot ax_{42}+0.357058\cdot ax_{43}-0.149771\cdot ax_{44}+\;0.405909\cdot ax_{45}-0.246462\cdot ax_{46}-0.307179\cdot ax_{47}+0.329967\cdot ax_{48}\right).$$

(A7) $$ax_{52}=\tanh\left(-0.10355+0.836911\cdot ax_{41}+0.902282\cdot ax_{42}+0.699127\cdot ax_{43}+0.00914586\cdot ax_{44}+\;0.853297\cdot ax_{45}-0.594561\cdot ax_{46}-0.475542\cdot ax_{47}+0.687024\cdot ax_{48}\right).$$

(A8) $$ax_{53}=\tanh\left(-0.00550484+0.0706381\cdot ax_{41}+1.37087\cdot ax_{42}-0.489375\cdot ax_{43}+0.766119\cdot ax_{44}-\;0.76727\cdot ax_{45}+0.0118196\cdot ax_{46}+0.34148\cdot ax_{47}+0.0277991\cdot ax_{48}\right).$$

(A9) $$ax_{54}=\tanh\left(0.0484762+0.207462\cdot ax_{41}-1.17439\cdot ax_{42}+0.599398\cdot ax_{43}+0.884247\cdot ax_{44}+\;1.09625\cdot ax_{45}-0.181395\cdot ax_{46}+0.544369\cdot ax_{47}-0.0457279\cdot ax_{48}\right).$$

(A10) $$ax_{55}=\tanh\left(0.0185739+0.114457\cdot ax_{41}+0.193903\cdot ax_{42}+0.266349\cdot ax_{43}+0.0349446\cdot ax_{44}+\;0.360525\cdot ax_{45}-0.0275735\cdot ax_{46}-0.114269\cdot ax_{47}-0.0457279\cdot ax_{48}\right).$$

(A11) $$ax_{56}=\tanh\left(0.162456-0.200818\cdot ax_{41}-0.111272\cdot ax_{42}-0.0271401\cdot ax_{43}+0.0839329\cdot ax_{44}\;+0.0074793\cdot ax_{45}+0.0810916\cdot ax_{46}+0.0938168\cdot ax_{47}-0.0128759\cdot ax_{48}\right).$$

(A12) $$ax_{41}=\tanh\left(0.304087-0.93554\cdot ax_{31}-0.332438\cdot ax_{32}-0.735927\cdot ax_{33}+0.114776\cdot ax_{34}-\;0.210384\cdot ax_{35}+0.156608\cdot ax_{36}-0.378972\cdot ax_{37}+0.203964\cdot ax_{38}-0.149654\cdot ax_{39}+0.431182\cdot \;ax_{310}\right).$$

(A13) $$ax_{42}=\tanh\left(0.237625-0.062685\cdot ax_{31}+0.492318\cdot ax_{32}+0.0000816928\cdot ax_{33}+0.393211\cdot ax_{34}+\;0.767934\cdot ax_{35}+0.246462\cdot ax_{36}-0.421343\cdot ax_{37}+0.223478\cdot ax_{38}-0.669725\cdot ax_{39}-1.02597\cdot \;ax_{310}\right).$$

(A14) $$ax_{43}=\tanh\left(-0.329438+0.0174092\cdot ax_{31}+0.65158\cdot ax_{32}+0.301201\cdot ax_{33}+0.675368\cdot ax_{34}+\;0.00703462\cdot ax_{35}+0.280539\cdot ax_{36}+0.502622\cdot ax_{37}-0.326401\cdot ax_{38}+0.570339\cdot ax_{39}+0.682099\cdot \;ax_{310}\right).$$

(A15) $$ax_{44}=\tanh\left(-0.133381-0.108074\cdot ax_{31}-0.411905\cdot ax_{32}+0.0102024\cdot ax_{33}-0.0754423\cdot ax_{34}+\;0.564025\cdot ax_{35}-0.0710046\cdot ax_{36}-0.121997\cdot ax_{37}-0.674816\cdot ax_{38}-0.363496\cdot ax_{39}+0.490389\cdot \;ax_{310}\right).$$

(A16) $$ax_{45}=\tanh\left(0.0712822+0.369538\cdot ax_{31}-0.982137\cdot ax_{32}+0.0487879\cdot ax_{33}-0.00170483\cdot ax_{34}-\;0.389693\cdot ax_{35}-0.96266\cdot ax_{36}+0.704598\cdot ax_{37}-0.555597\cdot ax_{38}+0.252501\cdot ax_{39}+0.713304\cdot \;ax_{310}\right).$$

(A17) $$ax_{46}=\tanh\left(0.165274+0.530625\cdot ax_{31}+0.000449785\cdot ax_{32}-0.268531\cdot ax_{33}+0.365822\cdot ax_{34}-\;0.239763\cdot ax_{35}+0.220901\cdot ax_{36}-0.233759\cdot ax_{37}-0.651051\cdot ax_{38}-0.329246\cdot ax_{39}-0.258471\cdot \;ax_{310}\right).$$

(A18) $$ax_{47}=\tanh\left(0.141672-0.0391891\cdot ax_{31}-0.0704693\cdot ax_{32}-0.175119\cdot ax_{33}+0.716787\cdot ax_{34}-\;0.146807\cdot ax_{35}-0.389877\cdot ax_{36}+0.246424\cdot ax_{37}+0.377645\cdot ax_{38}-0.0800747\cdot ax_{39}+0.682193\cdot \;ax_{310}\right).$$

(A19) $$ax_{48}=\tanh\left(0.167999+0.880357\cdot ax_{31}+0.0174599\cdot ax_{32}+0.203427\cdot ax_{33}-0.629281\cdot ax_{34}-\;0.484972\cdot ax_{35}-0.166586\cdot ax_{36}-0.303889\cdot ax_{37}-0.150484\cdot ax_{38}-0.38319\cdot ax_{39}+0.18877\cdot \;ax_{310}\right).$$

(A20) $$ax_{31}=\tanh\left(0.583476+0.257305\cdot ax_{21}+0.39606\cdot ax_{22}+0.625066\cdot ax_{23}-0.265241\cdot ax_{24}-\;0.59034\cdot ax_{25}-0.521324\cdot ax_{26}-0.278803\cdot ax_{27}+0.393518\cdot ax_{28}-0.715381\cdot ax_{29}-0.134194\cdot \;ax_{210}\right).$$

(A21) $$ax_{32}=\tanh\left(-0.4261+0.634804\cdot ax_{21}-0.121212\cdot ax_{22}-0.289991\cdot ax_{23}+0.609914\cdot ax_{24}+\;0.0439993\cdot ax_{25}-0.451075\cdot ax_{26}+0.334219\cdot ax_{27}-0.0891551\cdot ax_{28}-0.584922\cdot ax_{29}+0.306643\cdot \;ax_{210}\right).$$

(A22) $$ax_{33}=\tanh\left(0.10659-0.615507\cdot ax_{21}-0.0443891\cdot ax_{22}-0.0848592\cdot ax_{23}+0.456915\cdot ax_{24}+\;0.142345\cdot ax_{25}-0.439859\cdot ax_{26}+0.12741\cdot ax_{27}+0.202291\cdot ax_{28}-0.184254\cdot ax_{29}-0.466343\cdot \;ax_{210}\right).$$

(A23) $$ax_{34}=\tanh\left(-0.150688+0.0475739\cdot ax_{21}+0.411486\cdot ax_{22}-0.748264\cdot ax_{23}+0.0760448\cdot ax_{24}-\;0.00419205\cdot ax_{25}-0.269192\cdot ax_{26}-0.139757\cdot ax_{27}-0.481452\cdot ax_{28}-0.61357\cdot ax_{29}-0.51427\cdot \;ax_{210}\right).$$

(A24) $$ax_{35}=\tanh\left(0.257212+0.25639\cdot ax_{21}+0.277878\cdot ax_{22}+0.102443\cdot ax_{23}+0.666715\cdot ax_{24}-\;0.401726\cdot ax_{25}+0.281977\cdot ax_{26}-0.215321\cdot ax_{27}+0.303246\cdot ax_{28}-0.405421\cdot ax_{29}+\;0.000360402\cdot ax_{210}\right).$$

(A25) $$ax_{36}=\tanh\left(0.212833+0.403199\cdot ax_{21}+0.341386\cdot ax_{22}-0.293033\cdot ax_{23}+0.149875\cdot ax_{24}-\;0.0625897\cdot ax_{25}-0.104747\cdot ax_{26}+0.747322\cdot ax_{27}-0.0410873\cdot ax_{28}-0.381452\cdot ax_{29}+0.557865\cdot \;ax_{210}\right).$$

(A26) $$ax_{37}=\tanh\left(0.13612+0.070754\cdot ax_{21}-0.141931\cdot ax_{22}+0.317007\cdot ax_{23}+0.473061\cdot ax_{24}+\;0.971685\cdot ax_{25}+0.674186\cdot ax_{26}-0.117296\cdot ax_{27}+0.165819\cdot ax_{28}+0.0580616\cdot ax_{29}-0.549804\cdot \;ax_{210}\right).$$

(A27) $$ax_{38}=\tanh\left(-0.241159-0.150599\cdot ax_{21}-0.31649\cdot ax_{22}-1.04636\cdot ax_{23}+0.242558\cdot ax_{24}+\;0.241968\cdot ax_{25}-0.110343\cdot ax_{26}+0.158529\cdot ax_{27}-0.0196748\cdot ax_{28}+0.296961\cdot ax_{29}+0.184\cdot \;ax_{210}\right).$$

(A28) $$ax_{39}=\tanh\left(0.110104-0.820008\cdot ax_{21}-0.540616\cdot ax_{22}-0.263387\cdot ax_{23}+0.00700437\cdot ax_{24}-\;0.592159\cdot ax_{25}+0.27605\cdot ax_{26}-0.0406511\cdot ax_{27}-0.484756\cdot ax_{28}-0.426393\cdot ax_{29}-0.31372\cdot \;ax_{210}\right).$$

(A29) $$ax_{310}=\tanh\left(-0.127382-0.615599\cdot ax_{21}-0.281532\cdot ax_{22}+0.488731\cdot ax_{23}-0.730324\cdot ax_{24}+\;0.859829\cdot ax_{25}+0.45729\cdot ax_{26}-0.441791\cdot ax_{27}-0.616392\cdot ax_{28}-0.371053\cdot ax_{29}-0.603444\cdot \;ax_{210}\right).$$

(A30) $$ax_{21}=\tanh\left(-0.03503+1.17792\cdot ax_{11}+0.536391\cdot ax_{12}-0.694888\cdot ax_{13}+0.488167\cdot ax_{14}+\;0.724072\cdot ax_{15}-0.0434757\cdot ax_{16}+0.190797\cdot ax_{17}+0.316145\cdot ax_{18}+0.33833\cdot ax_{19}+0.224688\cdot \;ax_{110}\right).$$

(A31) $$ax_{22}=\tanh\left(0.219872-0.139292\cdot ax_{11}+0.498359\cdot ax_{12}+0.184706\cdot ax_{13}+0.506286\cdot ax_{14}-\;0.179573\cdot ax_{15}+0.177916\cdot ax_{16}-0.105241\cdot ax_{17}-0.461557\cdot ax_{18}+0.365369\cdot ax_{19}+0.341493\cdot \;ax_{110}\right).$$

(A32) $$ax_{23}=\tanh\left(0.441113+0.585713\cdot ax_{11}+0.710594\cdot ax_{12}+0.000187878\cdot ax_{13}+0.694946\cdot ax_{14}-\;0.0253969\cdot ax_{15}+0.0666956\cdot ax_{16}+0.78373\cdot ax_{17}-0.150309\cdot ax_{18}+0.0361135\cdot ax_{19}+0.529901\cdot \;ax_{110}\right).$$

(A33) $$ax_{24}=\tanh\left(0.277789+0.00996534\cdot ax_{11}+0.311853\cdot ax_{12}-0.515361\cdot ax_{13}-1.21524\cdot ax_{14}-\;0.483273\cdot ax_{15}+0.0458816\cdot ax_{16}+0.680251\cdot ax_{17}-0.47257\cdot ax_{18}-0.377953\cdot ax_{19}-0.0153472\cdot \;ax_{110}\right).$$

(A34) $$ax_{25}=\tanh\left(-0.0365425+0.000522626\cdot ax_{11}+0.186409\cdot ax_{12}+0.625137\cdot ax_{13}-1.29349\cdot ax_{14}+\;0.441413\cdot ax_{15}-0.654638\cdot ax_{16}+0.203844\cdot ax_{17}+0.186017\cdot ax_{18}+0.162006\cdot ax_{19}+0.335084\cdot \;ax_{110}\right).$$

(A35) $$ax_{26}=\tanh\left(-0.117201+0.131572\cdot ax_{11}-0.359282\cdot ax_{12}+0.389462\cdot ax_{13}+0.0835228\cdot ax_{14}+\;0.513271\cdot ax_{15}+0.0913045\cdot ax_{16}+1.07162\cdot ax_{17}-0.0790713\cdot ax_{18}-0.423777\cdot ax_{19}-0.258301\cdot \;ax_{110}\right).$$

(A36) $$ax_{27}=\tanh\left(-0.187318-0.593468\cdot ax_{11}-0.457257\cdot ax_{12}-0.218044\cdot ax_{13}+0.170376\cdot ax_{14}+\;0.230136\cdot ax_{15}-0.384798\cdot ax_{16}+0.0577948\cdot ax_{17}+0.11429\cdot ax_{18}+0.533959\cdot ax_{19}+0.244539\cdot \;ax_{110}\right).$$

(A37) $$ax_{28}=\tanh\left(-0.0126921-0.0590893\cdot ax_{11}-0.0584762\cdot ax_{12}+0.428384\cdot ax_{13}-0.162718\cdot ax_{14}-\;0.28943\cdot ax_{15}-0.165741\cdot ax_{16}+0.223617\cdot ax_{17}-0.6501\cdot ax_{18}+0.546167\cdot ax_{19}-0.57052\cdot ax_{110}\right).$$

(A38) $$ax_{29}=\tanh\left(0.04489-0.605727\cdot ax_{11}-0.00105592\cdot ax_{12}-0.507067\cdot ax_{13}-0.354351\cdot ax_{14}+\;0.727824\cdot ax_{15}+0.0357734\cdot ax_{16}+0.208872\cdot ax_{17}-0.277591\cdot ax_{18}+0.0822228\cdot ax_{19}-0.251631\cdot \;ax_{110}\right).$$

(A39) $$ax_{210}=\tanh\left(0.0274462-0.464155\cdot ax_{11}+0.335326\cdot ax_{12}-0.189354\cdot ax_{13}+0.00273104\cdot ax_{14}+\;0.138291\cdot ax_{15}+0.699764\cdot ax_{16}+0.378692\cdot ax_{17}+0.363206\cdot ax_{18}+0.698019\cdot ax_{19}-0.267254\cdot \;ax_{110}\right).$$

(A40) $$ax_{11}=\tanh\left(4.767351553463+0.00010018415828968\cdot C_{\mathrm{FO}}+0.0041259755901707\cdot W_{\mathrm{FO}}+\;0.000209184787522328\cdot FA_{\mathrm{FO}}+0.00147687126084511\cdot CA_{\mathrm{FO}}-0.170582379127354\cdot WC_{\mathrm{FO}}^{-1}-\;0.00739795485330284\cdot CS_{\mathrm{FO}}+0.0014713281157207\cdot C_{\mathrm{SO}}+0.00573830926470374\cdot W_{\mathrm{SO}}-\;0.00348658190095467\cdot FA_{\mathrm{SO}}-0.00375199793921971\cdot CA_{\mathrm{SO}}-0.271415999837499\cdot WC_{\mathrm{SO}}^{-1}+\;0.00269179297167401\cdot CS_{\mathrm{SO}}+0.000645907802623046\cdot C-0.00511651789903316\cdot W-\;0.00119068504591733\cdot FA+0.00121507017924426\cdot CA-0.194768583102371\cdot WC^{-1}\right).$$

(A41) $$ax_{12}=\tanh\left(-2.90937028957849+0.000513987509738448\cdot C_{\mathrm{FO}}+0.0104205287263435\cdot W_{\mathrm{FO}}-\;0.0000300236136823883\cdot FA_{\mathrm{FO}}+0.00193442295465733\cdot CA_{\mathrm{FO}}+0.0304831451994348\cdot WC_{\mathrm{FO}}^{-1}-\;0.00348102557161981\cdot CS_{\mathrm{FO}}-0.00117363708739863\cdot C_{\mathrm{SO}}+0.00426298901301358\cdot W_{\mathrm{SO}}-\;0.00060247532418286\cdot FA_{\mathrm{SO}}+0.000619551260777826\cdot CA_{\mathrm{SO}}+0.202375734386783\cdot WC_{\mathrm{SO}}^{-1}-\;0.000233978962466558\cdot CS_{\mathrm{SO}}-0.00038450344939621\cdot C+0.00544033538809217\cdot W-\;0.000295834802908707\cdot FA-0.001576885731436\cdot CA-0.437227409538789\cdot WC^{-1}\right).$$

(A42) $$ax_{13}=\tanh\left(-1.28397136623645-0.00265425528655059\cdot C_{\mathrm{FO}}+0.00134392831181661\cdot W_{\mathrm{FO}}+\;0.00187950192048241\cdot FA_{\mathrm{FO}}-0.000256323950062182\cdot CA_{\mathrm{FO}}+0.012594583425795\cdot WC_{\mathrm{FO}}^{-1}+\;0.0137268911005708\cdot CS_{\mathrm{FO}}-0.00153196251341365\cdot C_{\mathrm{SO}}-0.0065857765027207\cdot W_{\mathrm{SO}}+\;0.00208609505432554-FA_{\mathrm{SO}}-0.00000592967658050422\cdot CA_{\mathrm{SO}}-0.11595281342328\cdot WC_{\mathrm{SO}}^{-1}-\;0.0104040315572809\cdot CS_{\mathrm{SO}}+0.00196054736213827\cdot C+0.0102110804430631\cdot W+\;0.000620781117815345\cdot FA-0.00187136972051576\cdot CA-0.255163361753211\cdot WC^{-1}\right).$$

(A43) $$ax_{14}=\tanh\left(4.21395574600285-0.00105127459631726\cdot C_{\mathrm{FO}}-0.0112148941682978\cdot W_{\mathrm{FO}}+\;0.00163727698209709\cdot FA_{\mathrm{FO}}+0.000638406176270943\cdot CA_{\mathrm{FO}}+0.245800411366534\cdot WC_{\mathrm{FO}}^{-1}+\;0.0074057996976356\cdot CS_{\mathrm{FO}}-0.00233892425018069\cdot C_{\mathrm{SO}}+0.00377676692330639\cdot W_{\mathrm{SO}}-\;0.000934383939230747\cdot FA_{\mathrm{SO}}-0.00383296357061587\cdot CA_{\mathrm{SO}}+0.599324030373907\cdot WC_{\mathrm{SO}}^{-1}-\;0.00246137349085538\cdot CS_{\mathrm{SO}}-0.000522500768390411\cdot C-0.00430098689691885\cdot W-\;0.00200997561736293\cdot FA+0.00176580319743452\cdot CA-0.242934179812162\cdot WC^{-1}\right).$$

(A44) $$ax_{15}=\tanh\left(-1.6446570342455+0.000533587828047032\cdot C_{\mathrm{FO}}+0.000952303651589457\cdot W_{\mathrm{FO}}+\;0.000871116154702258\cdot FA_{\mathrm{FO}}-0.00000492904456363928\cdot CA_{\mathrm{FO}}+0.0360531074572857\cdot WC_{\mathrm{FO}}^{-1}-\;0.00992286147701841\cdot CS_{\mathrm{FO}}+0.001469808247159\cdot C_{\mathrm{SO}}+0.00642860151461661\cdot W_{\mathrm{SO}}-\;0.00271657540072071\cdot FA_{\mathrm{SO}}+0.000246865509647522\cdot CA_{\mathrm{SO}}+0.0279125858257541\cdot WC_{\mathrm{SO}}^{-1}+\;0.0133855714042725\cdot CS_{\mathrm{SO}}-0.000541184824066393\cdot C-0.00824577692013099\cdot W-\;0.00000397881537241383\cdot FA+0.00235002971670078\cdot CA-0.180135513126205\cdot WC^{-1}\right).$$

(A45) $$ax_{16}=\tanh\left(-3.84884812355591+0.00115407871985479\cdot C_{\mathrm{FO}}-0.00375105915008307\cdot W_{\mathrm{FO}}+\;0.000390252688951681\cdot FA_{\mathrm{FO}}+0.00221305190140043\cdot CA_{\mathrm{FO}}+0.327755674417453\cdot WC_{\mathrm{FO}}^{-1}+\;0.00166910211893054\cdot CS_{\mathrm{FO}}-0.000117013083312986\cdot C_{\mathrm{SO}}-0.010706630159067\cdot W_{\mathrm{SO}}+\;0.000537751740713538\cdot FA_{\mathrm{SO}}-0.000386509173984826\cdot CA_{\mathrm{SO}}+0.038443112623923\cdot WC_{\mathrm{SO}}^{-1}+\;0.00789947089570246\cdot CS_{\mathrm{SO}}-0.00163089480403489\cdot C+0.00485445960094249\cdot W+\;0.00174073824194078\cdot FA+0.00107642389772748\cdot CA-0.0903836711929193\cdot WC^{-1}\right).$$

(A46) $$ax_{17}=\tanh\left(-1.87654029183636+0.00140014344181078\cdot C_{\mathrm{FO}}-0.00531433362617444\cdot W_{\mathrm{FO}}-\;0.00413045250962745\cdot FA_{\mathrm{FO}}+0.00228016416194765\cdot CA_{\mathrm{FO}}-0.312440638205596\cdot WC_{\mathrm{FO}}^{-1}+\;0.00244581570673698\cdot CS_{\mathrm{FO}}-0.00140933248958429\cdot C_{\mathrm{SO}}+0.00161013791429313\cdot W_{\mathrm{SO}}+\;0.00171544141339727\cdot FA_{\mathrm{SO}}-0.000329656644298609\cdot CA_{\mathrm{SO}}-0.448421258516497\cdot WC_{\mathrm{SO}}^{-1}-\;0.00723447006861429\cdot CS_{\mathrm{SO}}+0.000353775031899132\cdot C-0.0017893761303401\cdot W+\;0.0018920172469581\cdot FA+0.00306888686044287\cdot CA-0.0131009807144161\cdot WC^{-1}\right).$$

(A47) $$ax_{18}=\tanh\left(7.30775875969612+0.0025578242040305\cdot C_{\mathrm{FO}}-0.00303262947215815\cdot W_{\mathrm{FO}}-\;0.00165574273119679\cdot FA_{\mathrm{FO}}-0.00306199296034884\cdot CA_{\mathrm{FO}}-0.156923718980124\cdot WC_{\mathrm{FO}}^{-1}+\;0.00137102269966526\cdot CS_{\mathrm{FO}}-0.000419742348687123\cdot C_{\mathrm{SO}}+0.0061626385341524\cdot W_{\mathrm{SO}}-\;0.00237717598480315\cdot FA_{\mathrm{SO}}-0.000972453336543304\cdot CA_{\mathrm{SO}}-0.190327552460012\cdot WC_{\mathrm{SO}}^{-1}-\;0.00783522799632837\cdot CS_{\mathrm{SO}}-0.000892188050113014\cdot C-0.0015154846950607\cdot W+\;0.000314135115650862\cdot FA+0.000748914790764869\cdot CA-0.184269602615267\cdot WC^{-1}\right).$$

(A48) $$ax_{19}=\tanh\left(2.06633738444408-0.00195515963988164\cdot C_{\mathrm{FO}}+0.00138958650164713\cdot W_{\mathrm{FO}}+\;0.00262801203222595\cdot FA_{\mathrm{FO}}-0.000536163722811296\cdot CA_{\mathrm{FO}}-0.188122517295472\cdot WC_{\mathrm{FO}}^{-1}+\;0.00407903424912999\cdot CS_{\mathrm{FO}}-0.000183834803792603\cdot C_{\mathrm{SO}}-0.00183731758403244\cdot W_{\mathrm{SO}}-\;0.000774757922991741\cdot FA_{\mathrm{SO}}+0.000574911771861783\cdot CA_{\mathrm{SO}}-0.100967264097832\cdot WC_{\mathrm{SO}}^{-1}-\;0.0083462680924114\cdot CS_{\mathrm{SO}}+0.000798708705572374\cdot C-0.00233339826228642\cdot W-\;0.00163577879701815\cdot FA-0.00020955799115713\cdot CA-0.28849090239718\cdot WC^{-1}\right).$$

(A49) $$ax_{110}=\tanh\left(-5.09651077352556+0.000206496906491233\cdot C_{\mathrm{FO}}+0.00444536475350176\cdot W_{\mathrm{FO}}+\;0.00109290014495785\cdot FA_{\mathrm{FO}}+0.00228266395774151\cdot CA_{\mathrm{FO}}+0.191531435179031\cdot WC_{\mathrm{FO}}^{-1}+\;0.000987346948074925\cdot CS_{\mathrm{FO}}+0.000289024996218767\cdot C_{\mathrm{SO}}+0.00254484992089329\cdot W_{\mathrm{SO}}+\;0.00133254035553042\cdot FA_{\mathrm{SO}}+0.0018234072550613\cdot CA_{\mathrm{SO}}+0.332154625352893\cdot WC_{\mathrm{SO}}^{-1}-\;0.00258354089455455\cdot CS_{\mathrm{SO}}+0.000785116534796457\cdot C-0.00462902690413978\cdot W-\;0.00163107840072543\cdot FA-0.000691117800517391\cdot CA-0.134078811558199\cdot WC^{-1}\right).$$

## Appendix B

Appendix B contains the gradings and fitting curves for the designed recipes. Recipe #1 designed according to the Bolomey method is presented in Figure A1, and that designed according to the Fuller method is presented in Figure A2. Recipe #2 designed according to the Bolomey method is presented in Figure A3, and that designed according to the Fuller method is presented in Figure A4. Recipe #3 designed according to the Bolomey method is presented in Figure A5, and that designed according to the Fuller method is presented in Figure A6. Recipe #4 designed according to the Bolomey method is presented in Figure A7, and that designed according to the Fuller method is presented in Figure A8.
